# Estimating the phylogeny and divergence times of primates using a supermatrix approach

**DOI:** 10.1186/1471-2148-9-259

**Published:** 2009-10-27

**Authors:** Helen J Chatterjee, Simon YW Ho, Ian Barnes, Colin Groves

**Affiliations:** 1 Research Department of Genetics, Evolution and Environment, University College London, London WC1E 6BT, UK; 2 Department of Zoology, University of Oxford, Oxford OX1 3PS, UK; 3 Centre for Macroevolution and Macroecology, Research School of Biology, Australian National University, Canberra, ACT 0200, Australia; 4 School of Biological Sciences, Royal Holloway University of London, Egham, Surrey, TW20 0EX, UK; 5 School of Archaeology and Anthropology, Australian National University, Canberra, ACT 0200, Australia

## Abstract

**Background:**

The primates are among the most broadly studied mammalian orders, with the published literature containing extensive analyses of their behavior, physiology, genetics and ecology. The importance of this group in medical and biological research is well appreciated, and explains the numerous molecular phylogenies that have been proposed for most primate families and genera. Composite estimates for the entire order have been infrequently attempted, with the last phylogenetic reconstruction spanning the full range of primate evolutionary relationships having been conducted over a decade ago.

**Results:**

To estimate the structure and tempo of primate evolutionary history, we employed Bayesian phylogenetic methods to analyze data supermatrices comprising 7 mitochondrial genes (6,138 nucleotides) from 219 species across 67 genera and 3 nuclear genes (2,157 nucleotides) from 26 genera. Many taxa were only partially represented, with an average of 3.95 and 5.43 mitochondrial genes per species and per genus, respectively, and 2.23 nuclear genes per genus. Our analyses of mitochondrial DNA place Tarsiiformes as the sister group of Strepsirrhini. Within Haplorrhini, we find support for the primary divergence of Pitheciidae in Platyrrhini, and our results suggest a sister grouping of African and non-African colobines within Colobinae and of Cercopithecini and Papionini within Cercopthecinae. Date estimates for nodes within each family and genus are presented, with estimates for key splits including: Strepsirrhini-Haplorrhini 64 million years ago (MYA), Lemuriformes-Lorisiformes 52 MYA, Platyrrhini-Catarrhini 43 MYA and Cercopithecoidea-Hominoidea 29 MYA.

**Conclusion:**

We present an up-to-date, comprehensive estimate of the structure and tempo of primate evolutionary history. Although considerable gaps remain in our knowledge of the primate phylogeny, increased data sampling, particularly from nuclear loci, will be able to provide further resolution.

## Background

The evolutionary relationships of our own order, Primates, have been of central interest since the birth of phylogenetic analysis. There has been consistent attention towards the relationships of primates to other mammals, with molecular and (more recently) morphological evidence providing strong support for the placement of Primates in the superorder Euarchontoglires [1-3]. Within Primates, the relationships within and between various families and genera continue to cause debate, despite the numerous molecular estimates of the phylogeny that have been presented over the past 10 to 15 years [4]. With increasing concerns over the extinction risks facing many primates, along with the recent publication of complete nuclear genomes from the chimpanzee [5] and rhesus macaque [6], there has been a resurgence of interest in resolving the evolutionary relationships amongst these diverse taxa [4,7].

In modern classifications, the order Primates comprises two suborders: Strepsirrhini (wet-nosed primates) and Haplorrhini (dry-nosed primates). This has not always been the case. One of the foremost debates in primate systematics has long concerned the position of tarsiers. Traditionally viewed as being more closely associated with lemurs and lorises, tarsiers were placed within a suborder Prosimii, under the gradistic view of primate taxonomy [8]. Modern taxonomic schemes generally recognize their closer affiliation with monkeys and apes, grouping them with Haplorrhini [9]. The majority of molecular evidence supports the latter grouping [4,10-13], although a large number of molecular studies still provide support for the Prosimii concept [14-18]. The question is succinctly reviewed by Yoder [19] and is further examined by Eizirik *et al*. [18]. There is now general agreement on the higher-level relationships within the two suborders [20], with Strepsirrhini comprising Lorisiformes (galagos and lorises) and the sister-pairing of the monophyletic Lemuriformes (lemurs) and Chiromyiformes (the aye-aye), and with Haplorrhini consisting of Platyrrhini (New World monkeys) and Catarrhini (apes and Old World monkeys). Within these groups, however, there are numerous disagreements over interfamilial relationships. Molecular evidence has sometimes favored Cheirogaleidae as sister group to Lemuridae, although current evidence suggests that the four lemuriform families (Lemuridae, Cheirogaleidae, Lepilemuridae and Indriidae) represent a four-way split, which may be real or may simply reflect a lack of resolution [4,21,22]. Within Haplorrhini, controversial taxonomic issues remain. The paraphyly of an all-encompassing Cebidae with respect to the tamarins and marmosets is widely recognized now [9,23,24], but the branching order of the major lineages is still questionable. Among the Old World monkeys, particularly within Colobinae, intergeneric relationships are still unclear.

The timescale of primate evolution has also been the subject of numerous molecular analyses over the past few decades [4,11,18,20,21,23-32]. Typically, divergence time estimates made using molecular phylogenetic approaches have supported a much more protracted timeframe for primate evolution than that suggested by the fossil record [27,33]. Inferring the age of the most recent common ancestor of all primates using molecular data has been of particular interest, owing to the poor understanding of early primate fossils and the contested affinity of Plesiadapiformes. The oldest unambiguous primate fossil is dated at 55 million years [34,35], whereas molecular estimates often place the common primate ancestor in excess of 80 million years ago (MYA) [4,18]. Estimates have varied with the reconstruction method employed and genetic loci used. In some instances this has resulted in considerably different date estimates; for example, Raaum *et al*. [29] recently dated the Cercopithecoidea-Hominoidea split at 23 MYA, whilst Yoder and Yang [27] and Steiper and Young [26] favored an older date of 30-40 MYA. This is further exemplified by Kumar *et al*. [36], who showed that both sampling method and calibration dates affect the confidence limits of the estimated timing of the human-chimpanzee divergence (calculated at 4.86 - 7.02 MYA, depending on the preferred date of the split between apes and Old World monkeys). Furthermore, previous estimates have been limited by the number and range of primate species, genera and families included in phylogenetic analyses, leaving certain groups (such as Tarsiidae and Daubentoniidae) in need of further study.

The task of estimating primate divergence times has been complicated by the presence of pronounced substitution rate heterogeneity among lineages, a phenomenon that has been of long-standing interest. For example, Goodman's 'hominoid slowdown' hypothesis was proposed in the early 1960s [37,38], and has recently been strongly supported by genomic studies [39,40]. Detailed analyses of primate sequences have revealed extensive departures from rate constancy in several other parts of the tree [4,18,27], calling for the employment of relaxed-clock methods that can explicitly accommodate rate heterogeneity among lineages [41,42].

While there may be consensus regarding relationships across the main primate clades, there is continued disagreement at the species, genus and even family levels. One of the primary challenges in primate molecular phylogenetics remains the issue that different markers support conflicting trees. Introgression between congeneric species, occasionally even between species in different (if closely related) genera, is an ever-present possibility, as is the origin of whole species by hybridization. The macaque example, as analyzed by Tosi *et al*. [43,44], serves as a warning.

Previous attempts to reconstruct the phylogeny of whole orders, even classes, have often used a "supertree" approach [11,30]. This method has a number of important weaknesses [45,46]; we also point out below that, in Primates, equating trees of different quality has produced some extremely misleading results. In this study, we draw together data from a number of mitochondrial and nuclear genes to construct data supermatrices, with a view to developing a consensus tree and estimating dates for key divergence events.

## Results and Discussion

This study represents a comprehensive phylogenetic study of the Order Primates, with regard to both taxonomic and gene coverage. This enables previous phylogenetic assessments of the order, which have been performed at smaller scales, to be placed into context. Previous estimates of rate heterogeneity and divergence dates, which have been raised in a piecemeal fashion for various primate clades, can now be examined on a wider scale. Below, we present and discuss the results obtained by Bayesian and maximum-likelihood analysis of three DNA data supermatrices (mitochondrial species-level, mitochondrial genus-level, and nuclear genus-level).

### BEAST analysis of mitochondrial sequence data

**Figure 1:**
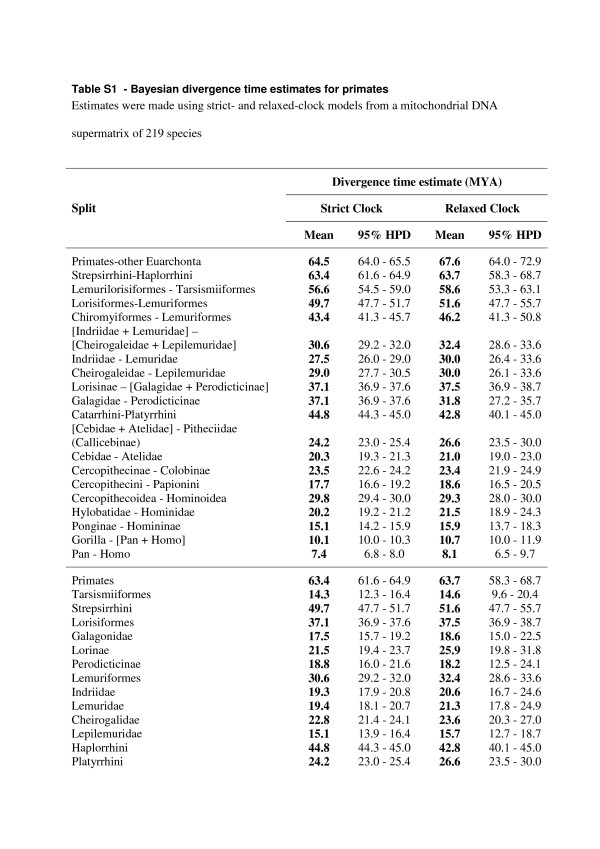
**Mitochondrial tree of primate genera**. Maximum-clade-credibility tree of the Order Primates, inferred from a genus-level mitochondrial DNA supermatrix using the Bayesian phylogenetic software *BEAST*. Nodes are labelled with a/b, where a represents the Bayesian posterior probability expressed as a percentage and b represents the percentage of 1,000 maximum-likelihood bootstrap replicates that support the node. Asterisks indicate 100% support; nodes with 100% support in both Bayesian and maximum-likelihood frameworks are labelled with single asterisks. The tree is drawn to a timescale, with node heights representing mean posterior estimates.

The results of our analyses using the Bayesian phylogenetic software *BEAST *[47], which is able to estimate the tree topology and divergence times in a relaxed-clock framework, are in agreement with those of previous studies regarding infra-ordinal relationships across the primates (Figure 1) [4,11,30]. The mean date estimate for the basal primate split, Strepsirrhini and Haplorrhini at 63.7 MYA (Additional file 1), is broadly in agreement with other estimates such as that of Goodman *et al*. [25] at 63 MYA, but younger than those of Steiper and Young [26] at 77.5 MYA and Janecka *et al*. [48] at 79.6 MYA.

#### Tarsiiformes

Our results place the tarsiers as sister group to Strepsirrhini, with a posterior probability of 1.0. The divergence date estimate for the split between tarsiers and strepsirrhines at 58.6 MYA is only slightly younger than the Primate-Euarchonta split (67.6 MYA) and the Strepsirrhini-Haplorrhini split (63.7 MYA). We concur with Eizirik *et al*. [18] that the split between the three major primate lineages occurred soon after the origin of primates, affording limited timed for a shared evolutionary history, which explains the difficulty in resolving the phylogenetic position of tarsiers. Given the long-standing disagreements over the phylogenetic position of tarsiers, this result obviously needs to be tested and retested. The consequences of a tarsiers/strepsirrhine association, if it is correct, are that haplorrhinism, including a haemochorial placenta and fovea and macula in the retina, are the primitive conditions for Primates as a whole. Given the general implications of this conclusion (for example, the retinal structure would require that the ancestral primates were diurnal), it would be wise to accumulate further molecular data. We also note that the very acceptance of the subordinal division between Strepsirrhini and Haplorrhini depends on the tarsiers being in a clade with the "anthropoids", not with the lemurs; were this association with the lemurs to be corroborated by future studies, the old category Prosimii would have to be revived.

#### Strepsirrhini

The phylogenetic analysis presented here is in agreement with most recent molecular studies which indicate that the major split within Strepsirrhini was between Lemuriformes/Chiromyiformes and Lorisiformes, although the split between Lemuriformes and Chiromyiformes came shortly afterward [4,21,22,30]. Furthermore, we find strong support for the placement of Cheirogaleidae within Lemuriformes, not within Lorisiformes.

Our mean estimate of the time to the most recent common ancestor of Lemuriformes, 32.4 MYA, is congruent with other date estimates [30], although notably younger than the date proposed by Matsui *et al*. [32] of 55.3 MYA. All molecular studies, including this one, support a very early divergence of *Daubentonia *from the other Malagasy lemurs, contra early morphological assessments (reviewed in [9]). It is mainly for this reason that Poux *et al*. [49] rejected the relevance of a putative land-bridge, which may have existed from the middle Eocene to the late Oligocene (about 45 to 26 MYA), to the question of the origin of the mammals of Madagascar. According to their molecular clock estimates, the tenrecs would have begun their diversification 31.8-19.7 MYA, the nesomyines (Malagasy rodents) 29.6-18.2 MYA, and the Malagasy carnivores 24.8-14.1 MYA, and all of these would more or less fit within the timeframe proposed for the supposed land-bridge. However, the Malagasy lemurs, whose initial diversification they dated to 69.6-51.6 MYA, would not. The initial diversification of Lemuriformes (*s.s*., that is, excluding *Daubentonia*) does fit within the timeframe for this land-bridge. If the Malagasy primates colonized via the land-bridge, they did so after the divergence of Lemuriformes and Chiromyiformes. Godinot [50] has pointed to similarities between *Daubentonia *and the enigmatic Fayûm primate *Plesiopithecus *and explicitly supported such a scenario. It is perhaps striking that the ancestors of Chiromyiformes and Lemuriformes separated so much longer ago than the known diversification within Lemuriformes (between one-and-a-half times and twice as long), particularly in light of the significant diversity of extant Lemuriformes. If the above scenario is correct and the common ancestors of Lemuriformes and Chiromyiformes arrived separately in Madagascar during the time of the putative land-bridge, it is evident that there is still much to learn about the time period between their Palaeocene or Eocene separation in Africa and the Late Oligocene when the simultaneous four-way split among the lemuriform families occurred in Madagascar.

Notwithstanding the above, it is clear that whether the most recent common ancestor of the Malagasy lemurs was Malagasy or African, the separation of the *Daubentonia *lineage followed very shortly after the separation between the Malagasy lemurs and Lorisiformes. Accordingly, we maintain the infraorder Chiromyiformes as separate from Lemuriformes.

Date estimates for the timing of the lorisiform radiation have varied widely, including: 13.8-14.2 MYA [4] and 55 MYA [51]. We propose a radiation at around 37.5 MYA which is, broadly speaking, compatible with the recent identification of one fossil from the Late Middle Eocene of Egypt as a galago and of another as a probable lorisid [52].

#### Strepsirrhini: Lemuriformes

**Figure 2:**
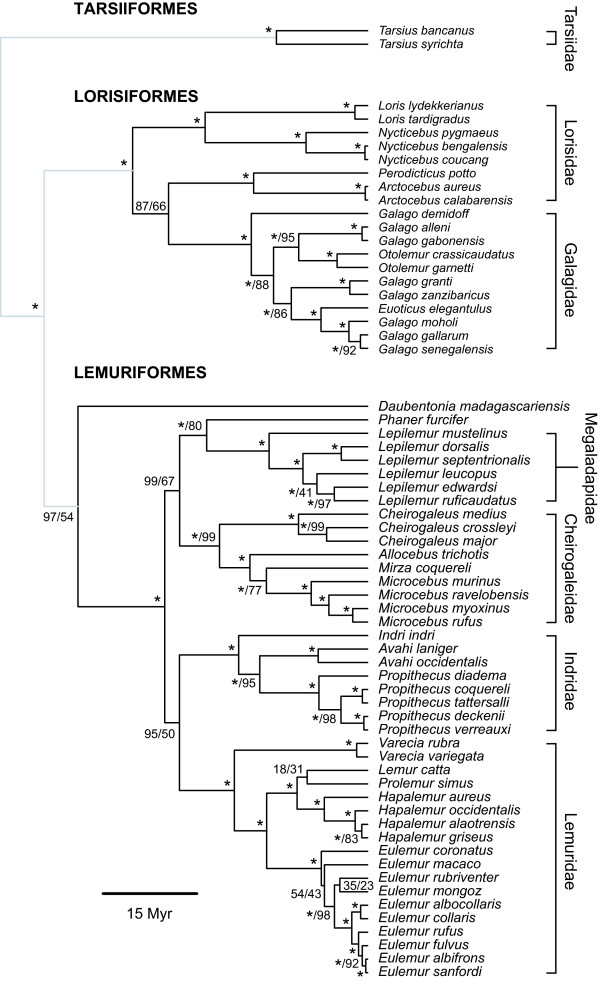
**Mitochondrial tree of strepsirrhine species**. Maximum-clade-credibility subtree of Strepsirrhini, inferred from a species-level mitochondrial DNA supermatrix using the Bayesian phylogenetic software *BEAST*. Nodes are labelled with a/b, where a represents the Bayesian posterior probability expressed as a percentage and b represents the percentage of 1,000 maximum-likelihood bootstrap replicates that support the node. Asterisks indicate 100% support; nodes with 100% support in both Bayesian and maximum-likelihood frameworks are labelled with single asterisks. The tree is drawn to a timescale, with node heights representing mean posterior estimates.

Inter-relationships amongst Lemuriform families are less well understood. Here we propose two sister clades, Lepilemuridae-Cheirogaleidae and Lemuridae-Indriidae (Figure 2); this is in contrast to previous studies which have either been unable to resolve relationships at this level at all, or provided other sister groupings for these families (compare the range of solutions proposed in [4,11,21,22]).

The grouping of *Phaner *with Lepilemuridae, rather than with the other Cheirogaleidae, is difficult to explain. At least one striking synapomorphy (the form of the carotid circulation) unites Cheirogaleidae. Potential explanations may include the following: the carotid synapomorphy may have characterized the common ancestor, but reversed in *Lepilemur*; the unusual carotid circulation may have evolved in parallel between *Phaner *and the (other) Cheirogaleidae, a possibility made more plausible by the fact that it recurs in the Lorisiformes; there could have been some parallelism in mitochondrial DNA sequences between *Phaner *and *Lepilemur*; there could have been introgression between lineages after their initial separation; or the unexpected placement could be an artefactual result caused by the presence of one or more nuclear pseudogenes in the data supermatrix. Roos *et al*. [53] also group *Phaner *with Lepilemuridae based on sequences of cytochome *b*, but this is in contrast to their analysis using SINEs in which *Phaner *groups with Cheirogaleidae. A further explanation may simply be that there are insufficient sequence data and that the true placement of *Phaner *will only be resolved when more data become available, although all assessments, except the supertree of Purvis [11], agree that it is by far the most phylogenetically distinct member of Cheirogaleidae. Within Cheirogaleidae (if *Phaner *is correctly excluded), the genus *Cheirogaleus *is depicted as the sister to the rest of the family (Figure 2), followed by *Allocebus*, leaving *Microcebus *and *Mirza *as sister genera. Divisions within both *Cheirogaleus *and *Microcebus *seem to be deep, implying a considerable time depth and raising the question of whether each should be split into two or more genera. The same question arises when considering the large genetic distance between *Lepilemur mustelinus *and other members of that genus.

Pastorini *et al*. [54] have examined inter-relationships amongst species of *Eulemur*. We find some agreement with these authors regarding the early divergence of *E. macaco *and of *E. coronatus *compared with the more recent divergence of *E. albifrons*, but other relationships are less consistent between the two studies. Complete congruence can be seen between the present results and Pastorini *et al*.'s [55] phylogeny of *Hapalemur *and its relatives. *Indri *is depicted as the sister to the other genera of the Indriidae; within *Propithecus*, we confirm that *P. tattersalli *belongs in the *P. verreauxi *group, as Pastorini *et al*. [55] maintained, not in the *P. diadema *group.

We have to inject some notes of caution into the interpretation of the rest of the lemuriform results. These relate to the accuracy of identifications. For example, prior to 2000, the small mouse lemur sympatric with *Microcebus murinus *in Kirindy was identified as *M. myoxinus*, but Rasoloarison *et al*. [56] showed that this identification was in error, and described the pygmy species as *M. berthae*. Likewise, until 2000, all Western Malagasy woolly lemurs were thought to belong to a single species, but Thalmann and Geissmann [57] began the process of splitting them into several species. We therefore cannot guarantee the correctness of the species determinations in these two genera.

#### Strepsirrhini: Lorisiformes

Inter-relationships amongst members of Lorisiformes have also been problematic, with little agreement reached across studies and genetic loci. The data are analytically challenging, as the results presented here illustrate (Figure 2). Within Lorisiformes, in our mitochondrial trees, Galagidae is monophyletic but Lorisidae is not - the two African genera, *Perodicticus *and *Arctocebus*, group with Galagidae rather than with the two Asian genera, *Loris *and *Nycticebus*. This supports the long-standing conclusions of Goodman (see [25] and elsewhere) that the African lorisids, Asian lorisids and galagids form a fairly even three-way split, and has implications for the polarity of their morphological adaptations (the "slow-climbing" features of lorisid anatomy would be plesiomorphic for Lorisiformes, and the vertical-clinging-and-leaping galagid anatomy would be derived from it). Three families, rather than two families, one of them having two subfamilies, would be the taxonomic consequence. So far, the only convincing evidence for monophyly of the family Lorisidae is the presence of three SINEs [53], though in one earlier study [11] not only was the family monophyletic, but an African/Asian partition within it did not exist - a reflection of the quality of the dataset on which it was based.

Within Galagidae, the tree shows paraphyletic relationships amongst what Groves [9] provisionally regarded as the genera *Galago*, *Otolemur *and *Euoticus*. The paraphyly of *Galago *with respect to *Otolemur *is not unexpected, and has already been espoused by Brandon-Jones *et al*. [58] and Masters *et al*. [59]. Groves [9] recognized *Otolemur *and *Euoticus *as genera separate from *Galago*, but considered that it would be "unsafe for the present" to recognize any others, distinctive though some of the species groups might be. It would appear from the present results that this potential paraphyly of *Galago *(*sensu *[9]) may have been underestimated. DelPero *et al*. [60] placed the species (better, species-group) commonly called *Galago alleni *in the *Otolemur *clade, and placed members of the *demidoff *and *zanzibaricus *groups as sisters to a clade combining *Galago *(the *senegalensis *group) and *Otolemur*. They did not, however, have any specimens of *Euoticus *or *Galago matschiei*, or of members of the "roller-caller" group (*G. orinus, G. rondoensis*) of Bearder *et al*. [61]; so their analysis, while suggestive, is incomplete. It is certain that there is much more to be learned about the inter-relationships of taxa in Galagidae, and a final taxonomic arrangement is not possible as yet; it seems likely that at least one new genus (for the *zanzibaricus *group) is needed, possibly one or two others (for the roller-callers and perhaps for *Galago matschiei*). Morphological studies are urgently needed to test this possibility and to define any new genera.

#### Haplorrhini

**Figure 3:**
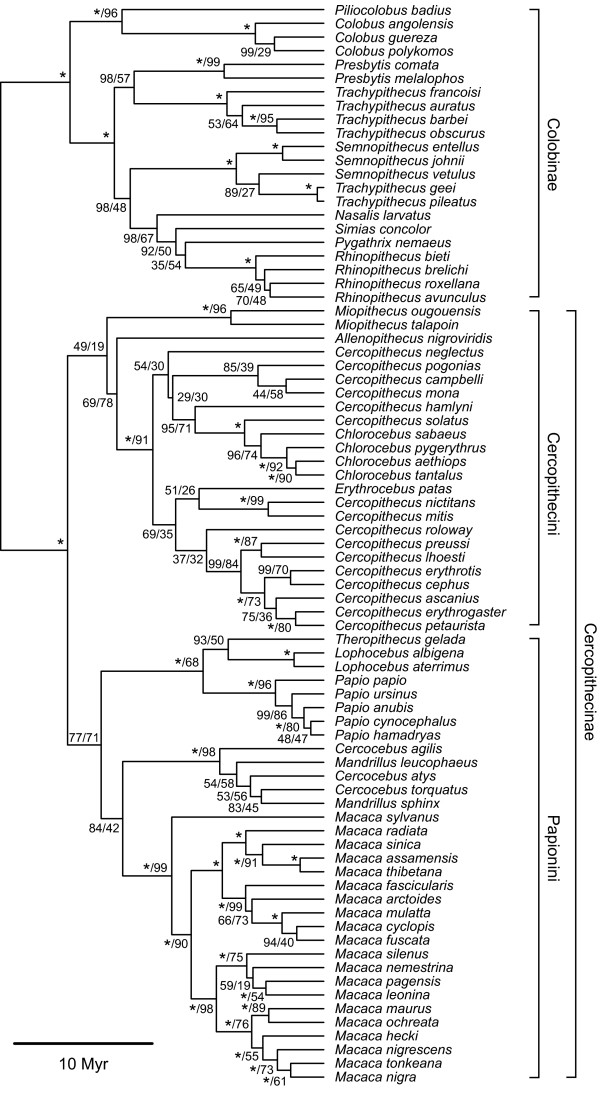
**Mitochondrial tree of cercopithecoid species**. Maximum-clade-credibility subtree of Cercopithecoidea, inferred from a species-level mitochondrial DNA supermatrix using the Bayesian phylogenetic software *BEAST*. Nodes are labelled with a/b, where a represents the Bayesian posterior probability expressed as a percentage and b represents the percentage of 1,000 maximum-likelihood bootstrap replicates that support the node. Asterisks indicate 100% support; nodes with 100% support in both Bayesian and maximum-likelihood frameworks are labelled with single asterisks. The tree is drawn to a timescale, with node heights representing mean posterior estimates.

**Figure 4:**
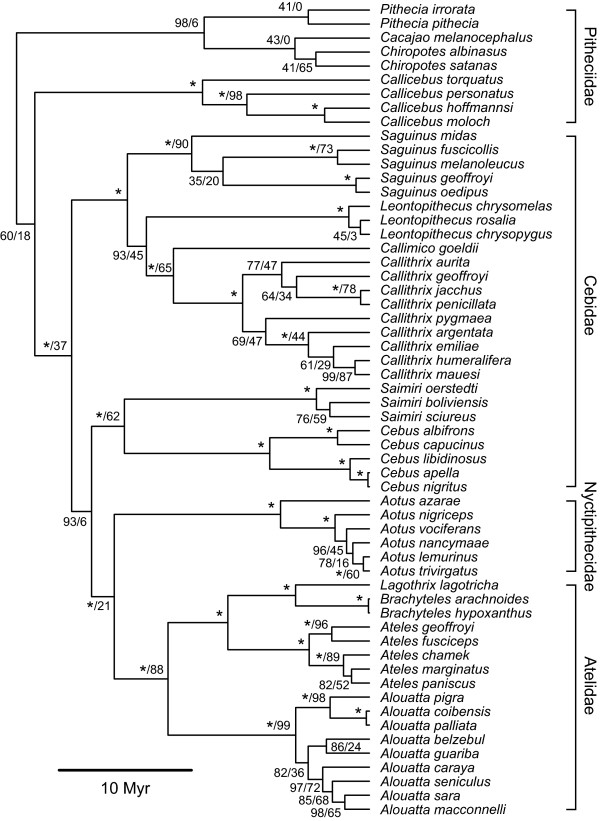
**Mitochondrial tree of platyrrhine species**. Maximum-clade-credibility subtree of Haplorrhini, inferred from a species-level mitochondrial DNA supermatrix using the Bayesian phylogenetic software *BEAST*. Nodes are labelled with a/b, where a represents the Bayesian posterior probability expressed as a percentage and b represents the percentage of 1,000 maximum-likelihood bootstrap replicates that support the node. Asterisks indicate 100% support; nodes with 100% support in both Bayesian and maximum-likelihood frameworks are labelled with single asterisks. The tree is drawn to a timescale, with node heights representing mean posterior estimates.

Within Haplorrhini, the catarrhine families Cercopithecidae, Hylobatidae and Hominidae are each monophyletic (Figure 3). Of the platyrrhines, however, only Atelidae and Aotidae are monophyletic, while Cebidae and Pitheciidae are both paraphyletic (Figure 4).

#### Haplorrhini: Platyrrhini

The platyrrhines have long been a source of debate regarding inter-relationships at family, genus and species levels. Platyrrhines were traditionally divided into two families, Callitrichidae (marmosets and tamarins) and Cebidae (cebids), sometimes even placing Goeldi's marmoset in a third family, Callimiconidae [62]. As long ago as 1981, however, this arrangement was challenged by Rosenberger [63], who pointed out its artificiality, and instead proposed to include the marmosets and tamarins in Cebidae, placing *Ateles *and its relatives together with *Pithecia *and its relatives in a second family, Atelidae (each of the two families having two subfamilies: Cebinae and Callitrichinae in Cebidae, and Atelinae and Pitheciinae in Atelidae). This perceptive analysis differed from modern molecular results only in one respect: that *Aotus *was included in Atelidae (in the subfamily Pitheciinae) instead of in a clade with Cebidae where we now know it belongs. Goodman *et al*. [25], Canavez *et al*. [64], Schneider *et al*. [23] and Poux and Douzery [4] agreed in aligning the marmosets with Cebinae and in placing *Ateles *and its relatives in a separate family Atelidae, but removed *Pithecia *and its relatives from that family and placed them in a separate family, Pitheciidae. Meireles *et al*. [65] demonstrated that, within the subfamily Atelinae, *Brachyteles *and *Lagothrix *form a sister group to *Ateles*, while *Alouatta *forms a sister group to Atelinae. Groves [9] adopted these divisions, but ill-advisedly replaced some of the family-group names with the others which have priority but in fact contravene the *International Code of Zoological Nomenclature *[66].

There are a number of questions remaining about Platyrrhini, such as the position of *Aotus*. For Groves [9], this genus forms a monotypic family, but for others, going back as far as Rosenberger [63], it is close to other groups among the platyrrhines. Do *Pithecia *and its relatives, *Callicebus*, *Chiropotes *and *Cacajao*, form a family on their own (Pitheciidae), or do they form a clade with *Ateles *and its relatives so that they should be included with them in Atelidae? How distinctive are the four groups of true marmosets: that is to say, should *Callimico, Cebuella *and *Callibella *be separated from *Callithrix *at generic level, or should they be retained in *Callithrix *as subgenera? What is the position of the yellow-tailed woolly monkey? The latter has commonly been included with other woolly monkeys as *Lagothrix flavicauda*, but Groves [9] could find no synapomorphic characters to unite them and on this basis revived the generic name *Oreonax *for *flavicauda*; no molecular sequence data are yet available for this species. Age estimates for the most recent common ancestor of Platyrrhini have ranged between 20.8 MYA [26] and 25 MYA [25]; here our mean estimate (26.6 MYA) is only marginally older. The question has a taxonomic importance quite apart from that of knowing the true phylogeny; Goodman *et al*. [25] proposed that taxonomic rank be linked to time of separation (approximately the Oligocene-Miocene boundary for families, and the Miocene-Pliocene boundary for genera).

Answers to some of these questions are suggested by the present study. In contrast to those studies that group *Aotus *within Cebidae [23,67,68], we find a closer affinity between *Aotus *and Atelidae, in part reviving Rosenberger's arrangement. The severe paucity of platyrrhine sequence data renders resolution of these issues problematic until data for a greater variety of species become available. Our date estimates for Cebidae, Atelidae and Pitheciidae are in broad agreement with previous estimates [23,25,26].

Some of the relationships between species are of particular interest here. In agreement with Goodman *et al*. [25], we place *Callicebus torquatus *as the sister taxon to the remaining congeneric species included in our analysis. Within *Saguinus*, there is no support for a division between a "bare-faced" (*geoffroyi*, *oedipus*) and a "hairy-faced" (*midas*, *fuscicollis*, *melanoleucus*); as with some of the lemuriform species, it is possible that incorrect identification of specimens, in particular *S. fuscicollis*, may have confounded analysis. Within *Callithrix*, we confirm a basic division between the Atlantic forest species (*C. jacchus *group) and the Amazonian species (including *C. pygmaea*, which until about 10 years ago was usually placed in a separate genus, *Cebuella *[11]). One consistent feature is the separation of Central American from South American groups: *Saimiri oerstedii *from *S. sciureus *and *boliviensis*; *Ateles geoffroyi *and *fusciceps *from other species; and *Alouatta pigra*, *coibensis *and *palliata *from the other congeneric species. The division within *Saguinus*, if it can be maintained, could be seen as part of the same biogeographic scenario.

#### Haplorrhini: Catarrhini

Within Catarrhini, our results agree with most other studies [20] that Hominidae and Hylobatidae form a sister clade to Cercopithecidae, and that, within Cercopithecidae, both Cercopithecinae and Colobinae are monophyletic (Figure 3). Phylogenetic relationships within these two subfamilies, especially Colobinae, are less well understood. Groves [9] divided the colobines into three informal groups based on geographic and morphological data: African (*Colobus, Procolobus *and *Piliocolobus*), Odd-Nosed (*Nasalis*, *Pygathrix*, *Rhinopithecus *and *Simias*), and Langurs (*Presbytis*, *Semnopithecus *and *Trachypithecus*). Here we find the Langurs and Odd-Nosed monkeys group together to the exclusion of the African group (*Colobus*). Xing *et al*. [69] found *Pygathrix *and *Nasalis *to form the sister group to *Trachypithecus*, followed by *Colobus*; this would suggest that the Langur and Odd-Nosed groups formed a sister clade to the African group. Here we find evidence for this sister grouping but no support for distinct Langur and Odd-Nosed sister clades; interspecific relationships among the Odd-Nosed colobines and Langurs have been little studied.

The problem of the South Asian langurs is a vexing one. Osterholz *et al*. [70] studied mitochondrial DNA, Y-chromosome DNA and retroposon integrations in *Semnopithecus *from North India, South India and Sri Lanka, the *Trachypithecus pileatus *group from the north-eastern part of the subcontinent, *T. vetulus *from Sri Lanka, *T. johnii *from South India, and a variety of *Trachypithecus *species from Southeast Asia. They found, as do we, that the Southeast Asian species cluster together regardless of the choice of genetic marker, but that the *T. pileatus *group formed a branch of the *Semnopithecus *clade for mitochondrial DNA whereas for Y-chromosome DNA it was part of the *Trachypithecus *clade, and for retroposon integrations it formed a branch equal to *Semnopithecus *and other *Trachypithecus *(lacking the integrations of either). Of the two southern *Trachypithecus *species, which are morphologically very similar and have generally been reckoned to be closely related to each other, for mitochondrial DNA *T. johnii *formed a clade with South Indian *Semnopithecus *and *T. vetulus *with Sri Lankan *Semnopithecus*, for Y-chromosome DNA the two formed equal branches with the three *Semnopithecus *branches, and they shared two retroposon insertions with *Semnopithecus*. Our mitochondrial DNA results are congruent with these, and the results taken altogether indicate a complex pattern of hybridization in the past, which resulted in the formation of what are evidently several species of hybrid origin.

The other subfamily, Cercopithecinae, also presents problems. Xing *et al*. [69], using *Alu *elements, provided support for the widely held view that there are two tribes within Cercopithecines, Papionini (*Macaca, Papio*, *Theropithecus*, *Lophocebus*, *Cercocebus *and *Mandrillus*) and Cercopithecini (*Allenopithecus*, *Miopithecus*, *Erythrocebus*, *Chlorocebus *and *Cercopithecus*); these findings are corroborated here. Date estimates for the Cercopithecini-Papionini split have tended to be more recent than the mean date proposed here (~18.6 MYA) generally being around 10 MYA [26,29]. There is some consensus [69] that *Macaca *forms a sister group to the rest of Papionini, with a baboon group of genera and a mandrill group of genera forming sister groups within this separate clade. We likewise find distinct baboon (*Papio*) and mandrill (*Mandrillus*) clades, but do not find support for the separate sister- group status of these clades relative to *Macaca*.

Divergence patterns within Cercopithecini are not completely resolved (Figure 3). In most schemes *Allenopithecus *is sister to the other genera (see, for example, [71]), but like Xing *et al*. [69], we find that the lineage leading to *Miopithecus *was the first to separate, although posterior probability support is only 0.69. *Allenopithecus*, the next lineage to separate form the remaining genera of Cercopithecini, is extremely morphologically different, retaining a considerable amount of symplesiomorph (papionin-like) conditions.

It is within Cercopithecini, subsequent to the separation of *Miopithecus *and *Allenopithecus*, that our results seem to be entirely novel and unexpected. Instead of a *Cercopithecus *clade contrasting with a *Chlorocebus/Erythrocebus/Allochrocebus *clade (arboreal and terrestrial clades of [71]), we have some *Cercopithecus *species groups (*neglectus, mona, hamlyni*) forming a clade with *Allochrocebus solatus *and *Chlorocebus*, while others (*mitis, diana, cephus*) form a clade with *Allochrocebus lhoesti *plus *preussi *and *Erythrocebus*. However, the posterior probabilities of groupings within Cercopithecini are generally low.

There have been numerous studies focusing on interspecific relationships amongst one of the most speciose and successful primate groups, the macaques, beginning with Fooden [72] and elsewhere). Fooden divided the genus into four species groups: the *sylvanus-silenus *group (including *nemestrina *and the Sulawesi macaques), the *fascicularis *group (including *mulatta *and others), the *sinica *group (including *assamensis *and others) and *Macaca arctoides *forming a group on its own. This initial division was based on morphology, in particular the shape of the penis. Molecular studies have tended to corroborate this, with the notable exception that there is a general consensus that the basal divergence within the macaques was between *Macaca sylvanus *and the Asian species, the association between *M. sylvanus *and Asian macaques like *M. silenus *being based on symplesiomorphic states [43,73,74]. We provide further evidence for the basal divergence of *M. sylvanus *here. Tosi *et al*. [73] and Evans *et al*. [75] recovered three primary clades, corresponding to the *silenus, sinica *and *fascicularis *groups. In their study of the *silenus *group, the Sulawesi macaques in particular, Evans *et al*. [75] suggested that *M. hecki *and *M. ochreata *are sister taxa to another clade (*M. tonkeana, M. nigrescens*, *M. nigra *and *M. maura*), which might have had separate origins outside Sulawesi; we however recover a sister grouping of distinct *silenus *and Sulawesi clades. We, like others, find a distinct *sinica *group (*M. radiata, M. sinica, M. assamensis *and *M. thibetana*), and a *fascicularis *group encompassing *M. fascicularis, M. arctoides, M. mulatta*, *M. cyclopis *and *M. fuscata*. With the current mitochondrial dataset we are unable to test the hypothesis of Tosi *et al*. [43] that *Macaca arctoides *is a species of hybrid origin between early members of the *fascicularis *and *sinica *groups: our mitochondrial analysis is in accord with theirs in resembling the *fascicularis *group, but we have no additional Y-chromosome DNA sequences, which would be important to verify the placement of *M. arctoides*.

**Figure 5:**
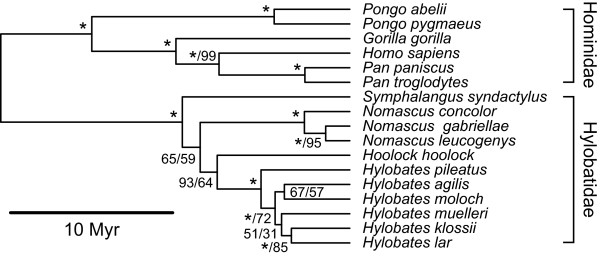
**Mitochondrial tree of hominoid species**. Maximum-clade-credibility subtree of Hominoidea, inferred from a species-level mitochondrial DNA supermatrix using the Bayesian phylogenetic software *BEAST*. Nodes are labelled with a/b, where a represents the Bayesian posterior probability expressed as a percentage and b represents the percentage of 1,000 maximum-likelihood bootstrap replicates that support the node. Asterisks indicate 100% support; nodes with 100% support in both Bayesian and maximum-likelihood frameworks are labelled with single asterisks. The tree is drawn to a timescale, with node heights representing mean posterior estimates.

Within Hominoidea (Figure 5) there is general consensus that Hylobatidae, followed by *Pongo*, then *Gorilla *and finally *Pan *and *Homo *as sister taxa, represent the pattern of divergence (see, most recently, [29]). The family Hylobatidae has recently received considerable attention with a general consensus being reached regarding its taxonomy which includes four genera: *Hylobates *(*H. lar*, *H. pileatus*, *H. agilis*, *H. albibarbis*, *H. moloch*, *H. muelleri *and *H. klossii*), *Hoolock *(*H. hoolock *and *H. leuconedys*), *Nomascus *(*N. concolor*, *N. nasutus*, *N. gabriellae*, *N. leucogenys*, *N. siki *and *N. hainanus*) and *Symphalangus *(*S. syndactylus*) [9,58,76-78], and there is now overwhelming support for the monophyly of each of the four genera [79-84]. There is some consensus that *Nomascus *represents the first to group to diverge, followed by *Symphalangus*, leaving *Hoolock *and *Hylobates *as sister genera [76,81,82] (but see also [11], which had *Symphalangus *and *Nomascus *forming a polytomy with the rest of Hylobatidae), Takacs *et al*. [83] disagreed with Roos and Geissmann [76] and Chatterjee [81,82], finding that *Hoolock *represented the first to diverge, with *Nomascus *as a sister group to *Hylobates*. Here we find support for a (*Symphalangus*,(*Nomascus*,(*Hoolock*, *Hylobates*))) divergence pattern, differing from Roos and Geissmann [76] and Chatterjee [81,82] only in the position of *Nomascus*. Our mean date estimate for the hylobatid clade (10.3 MYA) is in agreement with previous estimates [81,82,84,85].

Cercopithecoid-Hominoid divergence time estimates have ranged from 20 MYA [86], based on the premise of a global molecular clock) to 47 MYA [87], based on a calibration outside the order Primates), with most estimates being around 30 MYA [26,31,32,48]; our estimate of 29.3 MYA is congruent with recent estimates. Our date estimate for the Hylobatidae-Hominidae split (21.5 MYA) is in broad agreement with those of Hasegawa *et al*. [31] (21.7 MYA) and Matsui *et al*. [32] (19.9 MYA), but somewhat older than those proposed by Goodman *et al*. [25] at 18MYA, Yoder and Yang [27] at 11-17 MYA and Raaum *et al*. [29] at 16.8 MYA. Estimated dates for other splits within the Hominidae are consistent with those proposed by Yoder and Yang [27], Hasegawa *et al*. [31], Raaum *et al*. [29], Steiper and Young [26] and Matsui *et al*. [32]. One explanation for this disparity could be the use of multiple calibration points employed here. Previous studies have tended to employ a small number of calibration points based on interval age ranges for fossils. Raaum *et al*. [29], for example, employed three calibration points determined by combining fossil dates within an age range interval and assuming a median within that range. Their Cercopithecoid-Hominoid date estimate of 23 MYA is an estimate based on several hominoid fossils including *Proconsul *(dated to 19-20 MYA) and *Kamoyapithecus *(dated to 24-28 MYA), plus the earliest specimen of the stem cercopithecoid *Victoriapithecus *(dated to 19 MYA). Whilst reliable paleontological specimens have been invoked to produce the lineage divergence estimates, some important integral dating information has inevitably been lost. Here we have attempted to circumvent this issue by using calibration bounds based on multiple fossil data across the whole phylogeny.

### *MrBayes *Analysis of Mitochondrial Sequence Data

Comparison of the *BEAST *results against those produced by *MrBayes *shows that there is considerable congruence between the two forms of Bayesian phylogenetic analysis, at a number of taxonomic levels (Additional files 2 and 3). The trees estimated from the mitochondrial data are in general agreement, although inconsistencies can be seen at species-level within Lemuriformes, Platyrrhini and Cercopithecidae. However, these relate to nodes with relatively low support.

### Maximum-likelihood Analysis of Mitochondrial Sequence Data

Maximum-likelihood support for the Bayesian maximum-clade-credibility trees was estimated using 1,000 bootstrap replicates. For most nodes in the mitochondrial trees, the level of maximum-likelihood bootstrap support was lower than the posterior probability obtained using Bayesian analysis. For a small number of nodes, notably within Pitheciidae, there was no maximum-likelihood support for the nodes estimated using Bayesian analysis. However, these conflicts were limited to nodes with low posterior probabilities. Within Strepsirrhines, the inferred phylogenetic relationships received strong support under both methodological frameworks.

### Analyses of Nuclear Sequence Data

**Figure 6:**
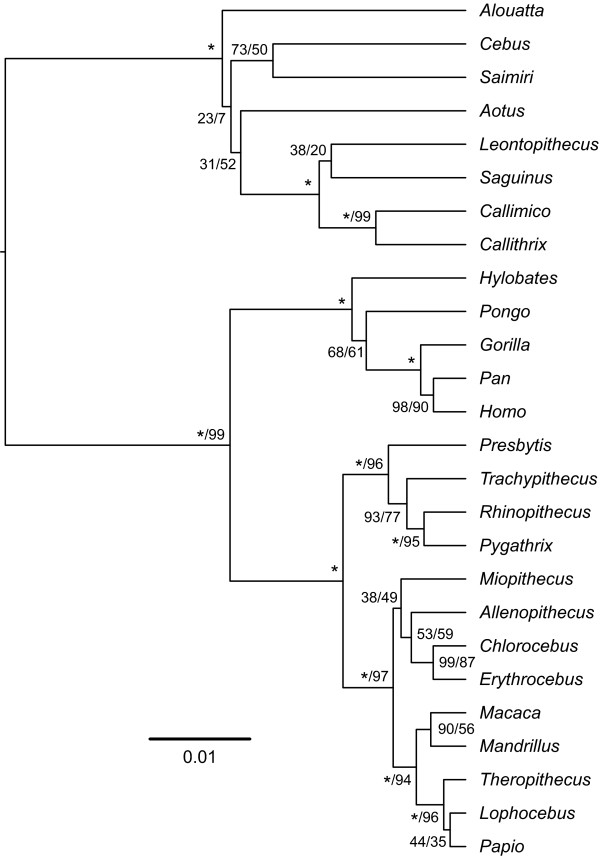
**Nuclear tree of haplorrhine genera**. Maximum-clade-credibility tree of Haplorrhini, inferred from a genus-level nuclear DNA supermatrix using the Bayesian phylogenetic software *BEAST*. Nodes are labelled with a/b, where a represents the Bayesian posterior probability expressed as a percentage and b represents the percentage of 1,000 maximum-likelihood bootstrap replicates that support the node. Asterisks indicate 100% support; nodes with 100% support in both Bayesian and maximum-likelihood frameworks are labelled with single asterisks. The tree is drawn to an arbitrary timescale, obtained using a fixed substitution rate of 1.0 substitution/site/time-unit. Node heights represent mean posterior estimates.

Taxonomic coverage of nuclear genes across the order was very poor, with almost no nuclear sequence data for strepsirrhines. Consequently it would not have been possible to combine the mitochondrial and nuclear sequences into a single supermatrix. Instead, a separate analysis of a nuclear data supermatrix, comprising three genes, was performed for haplorrhines. As indicated by the limited availability of nuclear sequence data on GenBank, there have been relatively few studies assessing primate phylogenetic interrelationships using nuclear DNA sequences and no large scale cross-taxic studies, as attempted here. The results of our study (Figure 6) agree with the mitochondrial analysis presented here, with the main inter-generic relationships remaining the same in both trees. Likewise the *MrBayes *analysis of the nuclear data (Additional file 4) is congruent with the *BEAST *nuclear and mitochondrial genus-level analyses, whereas maximum-likelihood bootstrap support was relatively low for most nodes. We conclude that, at the level of analysis we have been able to achieve, there has been little or no indication of the sort of discrepancy between mitochondrial and nuclear DNA that might imply the origin of any clade by hybridisation.

### Substitution Rate Heterogeneity

Analysis using the uncorrelated relaxed-clock model in *BEAST *provided insights into several characteristics of substitution rate heterogeneity among lineages. In the species-level analysis of mitochondrial data, the coefficient of variation of rates was 0.265 with a 95% highest posterior density (HPD) interval of 0.233 - 0.303. This excludes 0, which is the expected value under the assumption of a global molecular clock. In the genus-level mitochondrial analysis, the coefficient of variation of rates was 0.297 (95% HPD interval: 0.245 - 0.353), again rejecting the assumption of a strict molecular clock. In contrast, a strict clock could not be rejected for the nuclear data.

### Divergence Date Estimates

Estimates of divergence dates across the main splits and nodes were similar using the strict- and relaxed-clock models in *BEAST*. The framework provided by this program was the most appealing because of its ability to incorporate various sources of error, due to the co-estimation of phylogeny and divergence times using a relaxed-clock. Estimating the tree topology in the presence of significant rate heterogeneity among lineages can be problematic, because the assumed model of rate variation can change the posterior probabilities of different trees [41]. The aforementioned characteristics of *BEAST*, along with its ability to assign separate substitution models to different data partitions, offer a substantial advantage over alternative dating methods such as those available in the software *r8s *[88]. Additional file 1 provides divergence date estimates obtained using the Bayesian relaxed clock in *BEAST*, with a sample of comparative estimates derived from previous studies, chosen on the basis that they present the widest coverage of species and splits, shown in Additional file 5.

## Conclusion

We present a comprehensive estimate of primate phylogeny using mitochondrial data; a similar nuclear analysis is not possible at present due to lack of sequence data. The multi-gene approach adopted in this study has afforded the opportunity to investigate phylogenetic inter-relationships amongst primates at a variety of taxonomic levels from species through to infraorders. Furthermore, the combined data and methods employed have provided a novel opportunity to tackle phylogenetic reconstruction, divergence date estimation and substitution rate heterogeneity.

This study offers a novel reconstruction of phylogenetic relationships across the whole of the Order Primates down to species level and significantly builds upon previous whole-order phylogenies. It provides substantial support for previous studies in a number of key areas including: the primary divergence of Lorisiformes within Strepsirrhini, the primary divergence of Pitheciidae in Platyrrhini, a sister grouping of African and non-African colobines within Colobinae and of Cercopithecini and Papionini within Cercopthecinae. Other advances include a better understanding of species-level relationships within the lemurs, macaques and gibbons, and estimates of divergence dates across the whole tree.

In contrast to many molecular studies, but in support of others, our analysis has grouped *Tarsius *with the Strepsirrhini. Whilst the majority of evidence supports a haplorrhine grouping for these taxa, the incongruence of some data demonstrates the uniqueness of these primates and the value of continued efforts to reassess phylogeny as new evidence and novel techniques become available. There are still significant gaps in our understanding of phylogenetic relationships within Lorisiformes and Platyrrhini; crucially a range of molecular sequence data is required for various species within these groups before a convincing resolution can be reached.

The use of data supermatrices in the present study offers several advantages over a supertree approach. Chief among these is the ability to co-estimate the phylogeny and divergence times. In a supertree framework, the estimation of divergence times is performed indirectly. In contrast, in a Bayesian relaxed-clock framework, all divergence dates are estimated from the primary sequence data. This leads to a more realistic assessment of the uncertainty associated with date estimates, particularly when the analysis is performed using a relaxed-clock model with multiple calibrations. In turn, knowledge of branch-specific substitution rates and calibration bounds can inform phylogenetic reconstruction [41]. Other disadvantages of supertree methods, as discussed by Bininda-Emonds [46], also apply to the present study.

A few apparent inconsistencies are present in the inferred tree, including the failure to achieve reciprocal monophyly for some recently diverged clades. In the case of the mitochondrial sequence data, this could be due to the use of an effectively single locus, which heightens the risk of incomplete lineage sorting, a situation in which a gene tree is incongruent with the species phylogeny. The paucity of nuclear sequence data should be surmounted in the near future, with the increasing availability of sequences for multiple loci and even complete genomes [7]. Nuclear sequences, which evolve more slowly than the mitochondrial genome, could also increase the signal to noise ratio, leading to an improvement in the resolution of deeper primate relationships including final agreement regarding the placement of tarsiers.

## Methods

### Data Set

Published nucleotide sequences for seven mitochondrial genes (12s rRNA, 16s rRNA, *COII*, *CYTB*, *NADH3*, *NADH4L*, and *NDH4*) and three nuclear genes (*CXCR4*, *SRY*, and *TSPY*) were obtained from GenBank. These loci were chosen on the basis of taxonomic coverage; other candidate loci were discarded because of poor representation. In instances where a subspecies had recently been elevated to species level (such as within Hylobatidae) the most recent names were adopted and for the most part the taxonomy presented by Groves [9] was followed. The choice of outgroup, a flying lemur (*Cynocephalus variegatus*), was made in reference to previous studies into the placental mammal phylogeny [1,4].

Two approaches were employed to minimize the presence of nuclear copies of mitochondrial genes (numts) in the data. First, all of the protein-coding mitochondrial genes were translated into amino acids to check for the presence of stop codons. This measure has the potential to identify pseudogenes, but might not detect younger numts that have accumulated few mutations. Second, a neighbor-joining tree was constructed from each of the mitochondrial genes to identify any sequences that displayed unusual phylogenetic placements (e.g. sequences placed outside their expected infraorders). Despite their complete linkage, different mitochondrial genes can support mutually incompatible trees (for recent mammalian examples, see [89,90]), making it difficult to infer the presence of numts from phylogenetic incongruence. Thus, we cannot entirely discount the possibility that undetected numts have been included in the mitochondrial supermatrix.

Three data sets were assembled from the sequences available in GenBank: (i) species-level mitochondrial supermatrix; (ii) genus-level mitochondrial supermatrix; and (iii) genus-level nuclear supermatrix. These supermatrices are described below, and further details are given in Supplementary Information.

Sequences of each mitochondrial gene were first aligned using ClustalW 2.0.0 [91], then manually revised to remove poorly aligned regions of ambiguous homology. On average, the sequences of 3.95 mitochondrial genes were available for each species, with only 42 species being represented by all 7 mitochondrial genes analysed in this study. The gene alignments were concatenated to form a data matrix of 6,138 sites from 219 species (Additional file 6). The alignment was divided into four partitions: (i) first and second codon sites of protein-coding genes; (ii) third codon sites of protein-coding genes; (iii) stem regions of RNA genes; and (iv) loop regions of RNA genes. The stem and loop regions were determined with reference to the secondary structural models for *Homo sapiens *on the SILVA RNA database [92]. Substitution model selection was conducted for each of the four data partitions by comparison of Bayesian information criterion scores.

A second mitochondrial supermatrix was constructed, in which there was only a single representative of each genus. For some genera, a chimaeric sequence was formed by concatenating sequences from two congeneric species (Additional file 6). The purpose of this was to increase the completeness of the data supermatrix, and was only done for genera with unambiguous monophyly (based on the analysis of the species-level data supermatrix). Genera with uncertain monophyly were omitted from the analysis. In the resulting supermatrix, each genus was represented by an average of 5.43 mitochondrial genes. Data partitions were the same as for the mitochondrial species-level supermatrix.

The nuclear sequences were aligned manually and concatenated to form a data supermatrix of 2,157 sites from 26 genera. For some genera, a chimaeric sequence was formed by concatenating sequences from two congeneric species (Additional file 6). On average, each genus was represented by 2.23 nuclear genes. The alignment was divided into four partitions: (i) first codon sites of protein-coding genes; (ii) second codon sites of protein-coding genes; (iii) third codon sites of protein-coding genes; and (iv) introns. Substitution model selection was conducted for each partition by comparison of Bayesian information criterion scores.

### Phylogenetic Analysis

Bayesian phylogenetic analysis was performed on the mitochondrial species-level supermatrix using two different approaches. In the first approach, the phylogeny was estimated using the unconstrained Felsenstein model implemented by *MrBayes *3.1 [93]. In the second approach, the phylogeny and divergence times were co-estimated using the software *BEAST *1.4.7 [47].

In the *MrBayes *analysis, substitution model parameters were unlinked across the four data partitions. Posterior distributions of parameters, including the tree, were approximated using Markov chain Monte Carlo (MCMC) sampling. Two independent MCMC analyses were run, each with one cold chain and three heated chains. Samples from the posterior were drawn every 10,000 steps over a total of 10,000,000 steps per MCMC run, following a discarded burn-in of 1,000,000 steps. The results of the two analyses were combined and checked using *Tracer *1.4 [94]. Convergence was assessed by comparison of the two runs, while the adequacy of mixing was investigated by checking whether the effective sample sizes of parameters exceeded 200. The maximum-clade-credibility tree was identified using *TreeLogAnalyser *in the *BEAST *software package.

In the *BEAST *analysis, a separate substitution model was assumed for each of the four data partitions. By using the uncorrelated lognormal relaxed-clock model [41], rates were allowed to vary among branches without the *a priori *assumption of autocorrelation between adjacent branches [95]. This model allows sampling of the coefficient of variation of rates, which reflects the degree of departure from a global clock. For the sake of comparison, the analysis was repeated with the assumption of a global molecular clock. In both cases, a Yule (pure-birth process) prior was placed on the tree. Posterior distributions of parameters, including the tree, were approximated by sampling from two independent MCMC analyses. Samples from the posterior were drawn every 10,000 steps over a total of 10,000,000 steps per MCMC run, following a discarded burn-in of 1,000,000 steps. The results of the two analyses were combined, with acceptable mixing and convergence to the stationary distribution checked using *Tracer*. Using *TreeLogAnalyser *in the *BEAST *software package, the maximum-clade-credibility tree topology identified and given mean node heights calculated from the posterior distribution of trees.

In order to calibrate the age estimates of evolutionary divergence events, it is necessary to import some form of information about absolute times. This can come in the form of paleontological or biogeographic information, independent molecular date estimates, or known ages of ancient DNA sequences (for a recent review, see [96]). In the present analysis, the fossil record was used to inform the specification of 11 minimum age constraints. Two further constraints were placed on the age of the root, which was given a minimum bound of 64 MYA and a maximum bound of 110 MYA. In addition, two calibrations were given as exponential priors on nodal ages, which appear to represent an appropriate reflection of paleontological uncertainty [35,96,97]. Fossil ages were taken from Hartwig [98]. Details of these calibrations and associated fossil evidence are given in Additional file 7.

The mitochondrial and nuclear genus-level supermatrices were also analyzed using the two Bayesian methods described above. The details of the analyses are the same as for the mitochondrial supermatrix, but divergence time estimation was not attempted using the nuclear data because of poor taxonomic representation and phylogenetic resolution.

Maximum-likelihood support was calculated for the trees inferred from the three data supermatrices. To estimate the level of support, 1,000 bootstrap replicates were analysed using RaXML [99]. The alignment was partitioned as for Bayesian analyses described above, but a GTR+I+G substitution model was applied to each partition. This model was found to provide a significantly better fit to the data than a GTR+G model. Levels of bootstrap support were mapped on to the maximum-clade-credibility trees obtained using the Bayesian approach implemented in *BEAST*.

## Authors' contributions

HJC conceived of the study, participated in its design and coordination and drafted the manuscript. SYWH collected sequence data and performed all phylogenetic analyses, input ideas on study design and helped to draft the manuscript. IB participated in the study design and undertook initial sequence alignments. CG undertook phylogenetic interpretation and helped to draft the manuscript. All authors read and approved the final manuscript.

## Supplementary Material

Additional file 1 **Table S1. Bayesian divergence time estimates for primates**. Estimates were made using strict- and relaxed-clock models from a mitochondrial DNA supermatrix of 219 species. Click here for file

Additional file 2 **Figure S1. Maximum-clade-credibility tree of Order Primates, inferred from a genus-level mitochondrial DNA supermatrix using the Bayesian phylogenetic software *MrBayes***. Internal nodes are labeled with posterior probabilities given as percentages, with asterisks indicating 100% support. Branch lengths are measured in substitutions per site. Click here for file

Additional file 3 **Figure S2. Maximum-clade-credibility tree of Order Primates, inferred from a species-level mitochondrial DNA supermatrix using the Bayesian phylogenetic software *MrBayes***. Internal nodes are labeled with posterior probabilities given as percentages, with asterisks indicating 100% support. Branch lengths are measured in substitutions per site. Click here for file

Additional file 4 **Figure S3. Maximum-clade-credibility tree of Order Primates, inferred from a genus-level nuclear DNA supermatrix using the Bayesian phylogenetic software *MrBayes***. Internal nodes are labeled with posterior probabilities given as percentages, with asterisks indicating 100% support. Branch lengths are measured in substitutions per site. Click here for file

Additional file 5 **Table S2. Primate divergence times estimated in previous studies**. From left to right, the columns present dates from Purvis (1995) [11], Goodman *et al*. (1998) [25], Hasegawa *et al*. (2003) [31], Poux and Douzery (2004) [4], Yoder and Yang (2000^† ^[27], 2004^‡ ^[21]) and Yoder *et al*. (1996*) [28], Eizirik *et al*. (2004) [18], Raaum *et al*. (2005) [29], Steiper and Young (2006) [26], Schrago (2007) [24], Bininda-Emonds *et al*. (2007) [30], Janeèka *et al*. (2007) [48], and Matsui *et al*. (2009) [32]. Click here for file

Additional file 6 **Tables S3 S4 S5**. Table S3. Details of mitochondrial species-level supermatrix. Table S4. Details of mitochondrial genus-level supermatrix. Table S5. Details of nuclear genus-level supermatrix. Click here for file

Additional file 7 **Table S6. Primate fossil evidence used to calibrate phylogenetic estimates of divergence times**. Times are taken from Hartwig (2002). The earliest fossil evidence for the base of each group is used to place a minimum age constraint on its parent node in the tree. Minimum and maximum age constraints of 64 to 110 MYA, respectively, were also specified for the root node (divergence between primates and flying lemur). Click here for file

## References

[B1] Springer MS, Stanhope MJ, Madsen O, de Jong WW (2004). Molecules consolidate the placental mammal tree. Trends Ecol Evol.

[B2] Kriegs JO, Churakov G, Kiefmann M, Jordan U, Brosius J, Schmitz J (2006). Retroposed elements as archives for the evolutionary history of placental mammals. PLoS Biol.

[B3] Wible JR, Rougier GW, Novacek MJ, Asher RJ (2007). Cretaceous eutherians and Laurasian origin for placental mammals near the K/T boundary. Nature.

[B4] Poux C, Douzery EJP (2004). Primate phylogeny, evolutionary rate variations, and divergence times: A contribution from the nuclear gene IRBP. Am J Phys Anthropol.

[B5] The Chimpanzee Sequencing and Analysis Consortium (2005). Initial sequencing of the chimpanzee genome and comparison with the human genome. Nature.

[B6] Rhesus Macaque Genome Sequencing and Analysis Consortium (2007). Evolutionary and biomedical insights from the rhesus macaque genome. Science.

[B7] Pennisi E (2007). Genomicists tackle the primate tree. Science.

[B8] Luckett WP, MacPhee RDE (1993). Developmental evidence from the fetal membranes for assessing Archontan relationships. Primates and Their Relatives in Phylogenetic Perspective.

[B9] Groves CP (2001). Primate Taxonomy.

[B10] Bailey WJ, Fitch DHA, Tagle DA, Czelusniak J, Slightom JL, Goodman M (1991). Molecular evolution of the psi-eta-globin gene locus: Gibbon phylogeny and the hominoid slowdown. Mol Biol Evol.

[B11] Purvis A (1995). A composite estimate of primate phylogeny. Phil Trans R Soc Lond B.

[B12] Amrine-Madsen H, Koepfli KP, Wayne RK, Springer MS (2003). A new phylogenetic marker, apolipoprotein B, provides compelling evidence for euthterian relationships. Molecular Phylogenetics and Evolution.

[B13] Schmitz J, Roos C, Zischler H (2005). Primate phylogeny: molecular evidence from retroposons. Cytogenetic and Genome Research.

[B14] Hayasaka K, Gojobori T, Horai S (1988). Molecular phylogeny and evolution of primate mitochondrial DNA. Mol Biol Evol.

[B15] Jaworski CJ (1995). A reassessment of mammalian alpha A-crystallin sequences using DNA sequencing: implications for anthropoid affinities of tarsier. J Mol Evol.

[B16] Bellefroid EJ, Marine JC, Matera AG, Bourguignon C, Desai T, Healy KC, Bray-Ward P, Martial JA, Ihle JN, Ward DC (1995). Emergence of the ZNF91 Kruppel-associated box-containing zinc finger gene family in the last common ancestor of Anthropoidea. Proc Natl Acad Sci USA.

[B17] Murphy WJ, Eizirik E, Johnson WE, Zhang Y-P, Ryder OA, O'Brien SJ (2001). Molecular phylogenetics and the origins of placental mammals. Nature.

[B18] Eizirik E, Murphy WJ, Springer MS, O'Brien SJ, Ross CF, Kay RF (2004). Molecular Phylogeny and Dating of Early Primate Divergences. Anthropoid Origins: New Visions.

[B19] Yoder AD, Gursky S (2003). The phylogenetic position of genus *Tarsius*: whose side are you on?. Tarsiers: Past, Present and Future.

[B20] Steiper ME, Young NM, Hedges SB, Kumar S (2009). Primates. Timetree of Life.

[B21] Yoder AD, Yang Z (2004). Divergence dates for Malagasy lemurs estimated from multiple gene loci: geological and evolutionary context. Mol Ecol.

[B22] Yoder AD (1998). Back to the future: A synthesis of strepsirrhine systematics. Evolutionary Anthropology.

[B23] Schneider I, Schneider H, Schneider MP, Silva A (2004). The prion protein and New World primate phylogeny. Genetics and Molecular Biology.

[B24] Schrago CG (2007). On the time scale of New World primate diversification. Am J Phys Anthropol.

[B25] Goodman M, Porter CA, Czelusniak J, Page SL, Schneider H, Shoshani J, Gunnell G, Groves CP (1998). Toward a phylogenetic classification of primates based on DNA evidence complemented by fossil evidence. Molecular Phylogenetics and Evolution.

[B26] Steiper ME, Young NM (2006). Primate molecular divergence dates. Molecular Phylogenetics and Evolution.

[B27] Yoder AD, Yang ZH (2000). Estimation of primate speciation dates using local molecular clocks. Mol Biol Evol.

[B28] Yoder AD, Vilgalys R, Ruvolo M (1996). Molecular evolutionary dynamics of cytochrome *b *in strepsirrhine primates: The phylogenetic significance of third-position transversions. Mol Biol Evol.

[B29] Raaum RL, Sterner KN, Noviello CM, Stewart CB, Disotell TR (2005). Catarrhine primate divergence dates estimated from complete mitochondrial genomes: concordance with fossil and nuclear DNA evidence. J Hum Evol.

[B30] Bininda-Emonds OR, Cardillo M, Jones KE, MacPhee RD, Beck RM, Grenyer R, Price SA, Vos RA, Gittleman JL, Purvis A (2007). The delayed rise of present-day mammals. Nature.

[B31] Hasegawa M, Thorne JL, Kishino H (2003). Time scale of eutherian evolution estimated without assuming a constant rate of molecular evolution. Genes Genet Syst.

[B32] Matsui A, Rakotondraparany F, Munechika I, Hasegawa M, Horai S (2009). Molecular phylogeny and evolution of prosimians based on complete sequences of mitochondrial DNAs. Gene.

[B33] Arnason U, Gullberg A, Janke A (1998). Molecular timing of primate divergences as estimated by two nonprimate calibration points. J Mol Evol.

[B34] Bloch JI, Boyer DM (2002). Grasping primate origins. Science.

[B35] Benton MJ, Donoghue PC (2007). Paleontological evidence to date the tree of life. Mol Biol Evol.

[B36] Kumar S, Filipski A, Swarna V, Walker A, Hedges SB (2005). Placing confidence limits on the molecular age of the human-chimpanzee divergence. Proc Natl Acad Sci USA.

[B37] Goodman M (1961). The role of immunologic differences in the phyletic development of human behaviour. Human Biology.

[B38] Goodman M (1962). Evolution of the immunologic species specificity of human serum proteins. Human Biology.

[B39] Steiper ME, Young NM, Sukarna TY (2004). Genomic data support the hominoid slowdown and an Early Oligocene estimate for the hominoid-cercopithecoid divergence. Proc Natl Acad Sci USA.

[B40] Kim S-H, Elango N, Warden C, Vigoda E, Yi SV (2006). Heterogeneous genomic molecular clocks in primates. PLoS Genet.

[B41] Drummond AJ, Ho SYW, Phillips MJ, Rambaut A (2006). Relaxed phylogenetics and dating with confidence. PLoS Biol.

[B42] Thorne JL, Kishino H, Painter IS (1998). Estimating the rate of evolution of the rate of molecular evolution. Mol Biol Evol.

[B43] Tosi AJ, Morales JC, Melnick DJ (2003). Paternal, maternal, and biparental molecular markers provide unique windows onto the evolutionary history of macaque monkeys. Evolution.

[B44] Tosi AJ, Morales JC, Melnick DJ (2000). Comparison of Y chromosome and mtDNA phylogenies leads to unique inferences of macaque evolutionary history. Molecular Phylogenetics and Evolution.

[B45] Gatesy J, Matthee C, DeSalle R, Hayashi C (2002). Resolution of a supertree/supermatrix paradox. Syst Biol.

[B46] Bininda-Emonds ORP (2004). The evolution of supertrees. Trends Ecol Evol.

[B47] Drummond AJ, Rambaut A (2007). BEAST: Bayesian evolutionary analysis by sampling trees. BMC Evol Biol.

[B48] Janecka JE, Miller W, Pringle TH, Wiens F, Zitzmann A, Helgen KM, Springer MS, Murphy WJ (2007). Molecular and genomic data identify the closest living relative of Primates. Science.

[B49] Poux C, Madsen O, Marquard E, Vieites DR, de Jong WW, Vences M (2005). Asynchronous colonisation of Madagascar by four endemic clades of primates, tenrecs, carnivores and rodents, as inferred from nuclear genes. Syst Biol.

[B50] Godinot M (2006). Lemuriform origins as viewed from the fossil record. Folia Primatologica.

[B51] Yoder AD, Cartmill M, Ruvolo M, Smith K (1996). Ancient single origin for Malagasy primates. Proc Natl Acad Sci USA.

[B52] Seiffert ER, Simons EL, Attia Y (2003). Fossil evidence for an ancient divergence of lorises and galagos. Nature.

[B53] Roos C, Schmitz J, Zischler H (2004). Primate jumping genes elucidate strepsirrhine phylogeny. Proc Natl Acad Sci USA.

[B54] Pastorini J, Forstner MRJ, Martin RD (2000). Relationships among brown lemurs (Eulemur fulvus) based on mitochondrial DNA sequences. Molecular Phylogenetics and Evolution.

[B55] Pastorini J, Forstner MRJ, Martin RD (2002). Phylogenetic relationships of gentle lemurs (*Hapalemur*). Evolutionary Anthropology.

[B56] Rasoloarison RM, Goodman SM, Ganzhorn JU (2000). Taxonomic revision of mouse lemurs (*Microcebus*) in the western portions of Madagascar. International Journal of Primatology.

[B57] Thalmann U, Geissmann T (2000). Distribution and geographic variation in the Western Woolly Lemur (*Avahi occidentalis*) with description of a new species (*A. unicolor*). International Journal of Primatology.

[B58] Brandon-Jones D, Eudey AA, Geissmann T, Groves CP, Melnick DJ, Morales JC, Shekelle M, Stewart C-B (2004). Asian primate classification. International Journal of Primatology.

[B59] Masters JC, Anthony NM, de Wit MJ, Mitchell A (2005). Reconstructing the evolutionary history of the Lorisidae using morphological, molecular, and geological data. Am J Phys Anthropol.

[B60] DelPero M, Pozzi L, Masters JC (2006). A composite molecular phylogeny of living lemuroid primates. Folia Primatologica.

[B61] Bearder SK, Honess PE, Ambrose L, Izard MK (1995). Species Diversity among Galagos with Special Reference to Mate Recognition. Creatures of the Dark: The Nocturnal Prosimians.

[B62] Hershkovitz P (1977). Living New World Monkeys.

[B63] Rosenberger AL, Mittermeier RA (1981). Systematics: The Higher Taxa. Ecology and Behaviour of Neotropical Primates.

[B64] Canavez FC, Moreira MAM, Ladasky JJ, Pissinatti A, Parham P, Seuánez HN (1999). Molecular phylogeny of New World Primates (Platyrrhini) based on b2-microglobulin DNA sequences. Molecular Phylogenetics and Evolution.

[B65] Meireles CM, Czelusniak J, Schneider MPC, Muniz JAPC, Brigido MC, Ferreira HS, Goodman M (1999). Molecular phylogeny of ateline New World monkeys (Platyrrhini, Atelinae) based on gamma-globin gene sequences: Evidence that *Brachyteles *is the sister group of *Lagothrix*. Molecular Phylogenetics and Evolution.

[B66] Brandon-Jones D, Groves CP (2002). Neotropical Primate family-group names replaced by Groves (2001) in contravention of Article 40 of the International Code of Zoological Nomenclature. Neotropical Primates.

[B67] Schneider H (2000). The current status of the New World monkey phylogeny. Anais da Academia Brasileira de Ciências.

[B68] Ray DA, Xing J, Hedges DJ, Hall MA, Laborde ME, Anders BA, White BR, Stoilova N, Fowlkes JD, Landry KE (2005). *Alu *insertion loci and platyrrhine primate phylogeny. Molecular Phylogenetics and Evolution.

[B69] Xing J, Wang H, Han K, Ray DA, Huang CH, Chemnick LG, Stewart CB, Disotell TR, Ryder OA, Batzer MA (2005). A mobile element based phylogeny of Old World monkeys. Molecular Phylogenetics and Evolution.

[B70] Osterholz M, Walter L, Roos C (2008). Phylogenetic position of the langur genera *Semnopithecus *and *Trachypithecus *among Asian colobines, and genus affiliations of their species groups. BMC Evol Biol.

[B71] Tosi AJ, Melnick DJ, Disotell TR (2004). Sex chromosome phylogenetics indicate a single transition to terrestriality in the guenons (tribe Cercopithecini). J Hum Evol.

[B72] Fooden J (1976). Provisional classification and key to the living species of macaques (Primates: Macaca). Folia Primatologica.

[B73] Tosi AJ, Morales JC, Melnick DJ (2002). Y-chromosome and mitochondrial markers in *Macaca fascicularis *indicate introgression with Indochinese *M. mulatta *and a biogeographic barrier in the Isthmus of Kra. International Journal of Primatology.

[B74] Roos C, Zieglerb T, Hodges KJ, Zischlera H, Abegg C (2003). Molecular phylogeny of Mentawai macaques: taxonomic and biogeographic implications. Molecular Phylogenetics and Evolution.

[B75] Evans BJ, Supriatna J, Andayani N, Melnick DJ (2003). Diversification of Sulawesi macaque monkeys: Decoupled evolution of mitochondrial and autosomal DNA. Evolution.

[B76] Roos C, Geissmann T (2001). Molecular phylogeny of the major hylobatid divisions. Molecular Phylogenetics and Evolution.

[B77] Geissmann T (2002). Taxonomy and evolution of gibbons. Evolutionary Anthropology.

[B78] Mootnick A, Groves CP (2005). A new generic name for the hoolock gibbon (Hylobatidae). International Journal of Primatology.

[B79] Hall LM, Jones DS, Wood BA (1996). Evolution of the gibbon subgenera inferred from cytochrome b DNA sequence data. Molecular Phylogenetics and Evolution.

[B80] Zhang YP (1997). Mitochondrial DNA sequence evolution and phylogenetic relationships of gibbons. Acta Genetica Sinica.

[B81] Chatterjee HJ (2001). Phylogeny and biogeography of gibbons, genus *Hylobates*. PhD thesis.

[B82] Chatterjee HJ (2006). Phylogeny and biogeography of gibbons: A dispersal-vicariance analysis. International Journal of Primatology.

[B83] Takacs Z, Morales JC, Geissmann T, Melnick DJ (2005). A complete species-level phylogeny of the Hylobatidae based on mitochondrial ND3-ND4 gene sequences. Molecular Phylogenetics and Evolution.

[B84] Zehr S, Ruvolo M, Heider J, Mootnick A (1996). Gibbon phylogeny inferred from mitochondrial DNA sequences. Am J Phys Anthropol.

[B85] Porter CA, Page SL, Czelusniak J, Schneider H, Schneider MPC, Sampaio I, Goodman M (1997). Phylogeny and evolution of selected primates as determined by sequences of the eta-globin locus and 5' flanking regions. International Journal of Primatology.

[B86] Easteal S, Herbert G (1997). Molecular evidence from the nuclear genome for the time frame of human evolution. J Mol Evol.

[B87] Arnason U, Janke A (2002). Mitogenomic analyses of eutherian relationships. Cytogenetic and Genome Research.

[B88] Sanderson MJ (2003). r8s: inferring absolute rates of molecular evolution and divergence times in the absence of a molecular clock. Bioinformatics.

[B89] Rohland N, Malaspinas A-S, Pollack JL, Slatkin M, Matheus P, Hofreiter M (2007). Proboscidean mitogenomics: Chronology and mode of elephant evolution using mastodon as outgroup. PLoS Biol.

[B90] Willerslev E, Gilbert MTP, Binladen J, Ho SYW, Campos PF, Ratan A, Tomsho LP, da Fonseca RR, Sher A, Kuznetsova TV (2009). Analysis of complete mitochondrial genomes from extinct and extant rhinoceroses reveals lack of phylogenetic resolution. BMC Evol Biol.

[B91] Larkin MA, Blackshields G, Brown NP, Chenna R, McGettigan PA, McWilliam H, Valentin F, Wallace IM, Wilm A, Lopez R (2007). Clustal W and Clustal X version 2.0. Bioinformatics.

[B92] Pruesse E, Quast C, Knittel K, Fuchs B, Ludwig W, Peplies J, Glöckner FO (2007). SILVA: a comprehensive online resource for quality checked and aligned ribosomal RNA sequence data compatible with ARB. Nucleic Acids Res.

[B93] Huelsenbeck JP, Ronquist F (2001). MRBAYES: Bayesian inference of phylogenetic trees. Bioinformatics.

[B94] Rambaut A, Drummond AJ (2004). Tracer.

[B95] Ho SYW (2009). An examination of phylogenetic models of substitution rate variation among lineages. Biol Lett.

[B96] Ho SYW, Phillips MJ (2009). Accounting for calibration uncertainty in phylogenetic estimation of evolutionary divergence times. Syst Biol.

[B97] Ho SYW (2007). Calibrating molecular estimates of substitution rates and divergence times in birds. J Avian Biol.

[B98] Hartwig WC (2002). The Primate Fossil Record.

[B99] Stamatakis A (2006). RAxML-VI-HPC: maximum likelihood-based phylogenetic analyses with thousands of taxa and mixed models. Bioinformatics.

