# Supplementary material for: Estimating the phylogeny and divergence times of primates using a supermatrix approach
Source: BMC Evol Biol. 2009 Oct 27;9:259. doi: 10.1186/1471-2148-9-259 (PMC2774700; doi:10.1186/1471-2148-9-259)
Supplement: Additional file 1 — Table S1. Bayesian divergence time estimates for primates. Estimates were made using strict- and relaxed-clock models from a mitochondrial DNA supermatrix of 219 species. [file 1471-2148-9-259-S1.DOC]

## Table S1 - Bayesian divergence time estimates for primates

Estimates were made using strict- and relaxed-clock models from a mitochondrial DNA supermatrix of 219 species

| Split | Divergence time estimate (MYA) | | | |
| --- | --- | --- | --- | --- |
|  | **Strict Clock** | | **Relaxed Clock** | |
|  | **Mean** | **95% HPD** | **Mean** | **95% HPD** |
| Primates-other Euarchonta | **64.5** | 64.0 - 65.5 | **67.6** | 64.0 - 72.9 |
| Strepsirrhini-Haplorrhini | **63.4** | 61.6 - 64.9 | **63.7** | 58.3 - 68.7 |
| Lemurilorisiformes - Tarsismiiformes | **56.6** | 54.5 - 59.0 | **58.6** | 53.3 - 63.1 |
| Lorisiformes-Lemuriformes | **49.7** | 47.7 - 51.7 | **51.6** | 47.7 - 55.7 |
| Chiromyiformes - Lemuriformes | **43.4** | 41.3 - 45.7 | **46.2** | 41.3 - 50.8 |
| [Indriidae + Lemuridae] –  [Cheirogaleidae + Lepilemuridae] | **30.6** | 29.2 - 32.0 | **32.4** | 28.6 - 33.6 |
| Indriidae - Lemuridae | **27.5** | 26.0 - 29.0 | **30.0** | 26.4 - 33.6 |
| Cheirogaleidae - Lepilemuridae | **29.0** | 27.7 - 30.5 | **30.0** | 26.1 - 33.6 |
| Lorisinae – [Galagidae + Perodicticinae] | **37.1** | 36.9 - 37.6 | **37.5** | 36.9 - 38.7 |
| Galagidae - Perodicticinae | **37.1** | 36.9 - 37.6 | **31.8** | 27.2 - 35.7 |
| Catarrhini-Platyrrhini | **44.8** | 44.3 - 45.0 | **42.8** | 40.1 - 45.0 |
| [Cebidae + Atelidae] - Pitheciidae (Callicebinae) | **24.2** | 23.0 - 25.4 | **26.6** | 23.5 - 30.0 |
| Cebidae - Atelidae | **20.3** | 19.3 - 21.3 | **21.0** | 19.0 - 23.0 |
| Cercopithecinae - Colobinae | **23.5** | 22.6 - 24.2 | **23.4** | 21.9 - 24.9 |
| Cercopithecini - Papionini | **17.7** | 16.6 - 19.2 | **18.6** | 16.5 - 20.5 |
| Cercopithecoidea - Hominoidea | **29.8** | 29.4 - 30.0 | **29.3** | 28.0 - 30.0 |
| Hylobatidae - Hominidae | **20.2** | 19.2 - 21.2 | **21.5** | 18.9 - 24.3 |
| Ponginae - Homininae | **15.1** | 14.2 - 15.9 | **15.9** | 13.7 - 18.3 |
| Gorilla - [Pan + Homo] | **10.1** | 10.0 - 10.3 | **10.7** | 10.0 - 11.9 |
| Pan - Homo | **7.4** | 6.8 - 8.0 | **8.1** | 6.5 - 9.7 |
| Primates | **63.4** | 61.6 - 64.9 | **63.7** | 58.3 - 68.7 |
| Tarsismiiformes | **14.3** | 12.3 - 16.4 | **14.6** | 9.6 - 20.4 |
| Strepsirrhini | **49.7** | 47.7 - 51.7 | **51.6** | 47.7 - 55.7 |
| Lorisiformes | **37.1** | 36.9 - 37.6 | **37.5** | 36.9 - 38.7 |
| Galagonidae | **17.5** | 15.7 - 19.2 | **18.6** | 15.0 - 22.5 |
| Lorinae | **21.5** | 19.4 - 23.7 | **25.9** | 19.8 - 31.8 |
| Perodicticinae | **18.8** | 16.0 - 21.6 | **18.2** | 12.5 - 24.1 |
| Lemuriformes | **30.6** | 29.2 - 32.0 | **32.4** | 28.6 - 33.6 |
| Indriidae | **19.3** | 17.9 - 20.8 | **20.6** | 16.7 - 24.6 |
| Lemuridae | **19.4** | 18.1 - 20.7 | **21.3** | 17.8 - 24.9 |
| Cheirogalidae | **22.8** | 21.4 - 24.1 | **23.6** | 20.3 - 27.0 |
| Lepilemuridae | **15.1** | 13.9 - 16.4 | **15.7** | 12.7 - 18.7 |
| Haplorrhini | **44.8** | 44.3 - 45.0 | **42.8** | 40.1 - 45.0 |
| Platyrrhini | **24.2** | 23.0 - 25.4 | **26.6** | 23.5 - 30.0 |
| Cebidae | **18.6** | 17.4 - 19.9 | **18.5** | 16.0 - 20.9 |
| Atelidae | **14.3** | 13.1 - 15.5 | **15.2** | 13.0 - 17.5 |
| Pitheciidae (Callicebinae) | **21.6** | 19.8 - 23.4 | **26.6** | 23.5 - 30.0 |
| Catarrhini | **29.8** | 29.4 - 30.0 | **29.3** | 28.0 - 30.0 |
| Cercopithecidae | **23.5** | 22.6 - 24.2 | **23.4** | 21.9 - 24.9 |
| Cercopithecidae (Papionini) | **15.6** | 14.7 - 16.4 | **16.1** | 14.4 - 17.7 |
| Cercopithecidae (Cercopithecini) | **14.8** | 12.9 - 17.1 | **15.7** | 12.5 - 17.9 |
| Cercopithecidae (Macaca) | **10.0** | 9.4 - 10.7 | **11.0** | 9.9 - 12.4 |
| Cercopithecidae (Colobinae) | **18.0** | 17.2 - 18.8 | 18.4 | 16.8 - 20.1 |
| Hominoidea | **20.2** | 19.2 - 21.2 | **21.5** | 18.9 - 24.3 |
| Hylobatidae | **9.2** | 8.5 - 9.8 | **10.3** | 8.6 - 12.3 |
| Hylobates | **5.0** | 4.6 - 5.4 | **5.5** | 4.5 - 6.5 |
| Nomascus | **2.6** | 2.0 - 3.2 | **2.8** | 1.9 - 3.8 |
| Hominidae | **15.1** | 14.2 - 15.9 | **15.9** | 13.7 - 18.3 |
| Pongo | **4.5** | 3.9 - 5.0 | **4.7** | 3.2 - 6.1 |
| Pan | **2.6** | 2.2 - 2.9 | **2.8** | 1.8 - 3.9 |
