# Supplementary material for: Estimating the phylogeny and divergence times of primates using a supermatrix approach
Source: BMC Evol Biol. 2009 Oct 27;9:259. doi: 10.1186/1471-2148-9-259 (PMC2774700; doi:10.1186/1471-2148-9-259)
Supplement: Additional file 5 — Table S2. Primate divergence times estimated in previous studies. From left to right, the columns present dates from Purvis (1995) [11], Goodman et al. (1998) [25], Hasegawa et al. (2003) [31], Poux and Douzery (2004) [4], Yoder and Yang (2000† [27], 2004‡ [21]) and Yoder et al. (1996*) [28], Eizirik et al. (2004) [18], Raaum et al. (2005) [29], Steiper and Young (2006) [26], Schrago (2007) [24], Bininda-Emonds et al. (2007) [30], Janeèka et al. (2007) [48], and Matsui et al. (2009) [32]. [file 1471-2148-9-259-S5.DOC]

## Table S2 - Primate divergence times estimated in previous studies

From left to right, the columns present dates from Purvis (1995) [11], Goodman *et al*. (1998) [25], Hasegawa *et al.* (2003) [31], Poux and Douzery (2004) [4], Yoder and Yang (2000**^†^** [27], 2004**^‡^** [21]) and Yoder *et al*. (1996*) [28], Eizirik *et al.* (2004) [18], Raaum *et al*. (2005) [29], Steiper and Young (2006) [26], Schrago (2007) [24], Bininda-Emonds *et al*. (2007) [30], Janečka *et al*. (2007) [48], and Matsui *et al.* (2009) [32].

|  | **Date estimates from previous studies (MYA)** | | | | | | | | | | | | |
| --- | --- | --- | --- | --- | --- | --- | --- | --- | --- | --- | --- | --- | --- |
| **Split** | **Present study** | **Ref.**  **11** | **Ref.**  **25** | **Ref.**  **31** | **Ref.**  **4** | **Refs.**  **21,**  **27,**  **28** | **Ref.**  **18** | **Ref.**  **29** | **Ref.**  **26** | **Ref.**  **24** | **Ref. 30** | **Ref.**  **48** | **Ref.**  **32** |
| Primates-Euarchonta | **67.6** |  |  |  | 64.8 |  | 82.8 |  |  |  |  | 87.9 |  |
| Strepsirrhini-Haplorrhini | **63.7** | 57.2 | 63 |  |  |  | 72.2 |  | 77.5 |  |  |  |  |
| Lorisiformes-Lemuriformes | **51.6** | 41.8 |  |  |  | 62* | 71.4 |  | 57.1 |  |  |  |  |
| Indridae - Lemuridae | **30.0** | 20 |  |  |  |  |  |  |  |  |  |  |  |
| Catarrhini-Platyrrhini | **42.8** | 39.9 |  |  |  |  |  |  | 42.9 | 40.2 |  | 41.7 |  |
| [Cebidae + Atelidae] - Pitheciidae (Callicebinae) | **26.6** |  |  |  |  |  |  |  | 20.8 |  |  |  |  |
| Cercopithecinae -Colobinae | **23.4** | 14.7 |  |  |  |  |  | 16.2 |  |  |  |  |  |
| Cercopithcini - Papionini | **18.6** | 9.6 |  |  |  |  |  | 11.6 | 9.9 |  |  |  |  |
| Cercopithecoidea - Hominoidea | **29.3** | 27.5 |  |  |  | 30 -40† |  | 23 | 30.5 |  |  | 26.8 |  |
| Hylobatidae - Hominidae | **21.5** | 17.6 |  |  |  | 11 - 17† |  | 16.8 |  |  |  |  |  |
| Ponginae - Homininae | **15.9** | 14.5 |  |  |  |  | 43.6 | 14 | 18.3 |  |  |  | 45.3 |
| Gorilla – [Pan + Homo] | **10.7** | 8.1 |  |  |  | 7 - 9† |  | 8.1 | 8.6 |  |  |  |  |
| Pan – Homo | **8.1** | 7 |  |  |  | 4 - 6† | 20.1 | 6 | 6.6 |  |  |  |  |
|  |  |  |  |  |  |  |  |  |  |  |  |  |  |
| Primates | **63.7** |  | 63 |  |  |  |  |  |  |  | 87.7 | 79.6 |  |
| Strepsirrhini | **51.6** | 41.8 | 50 |  | 45.4 – 46.7 |  |  |  |  |  |  | 62.1 |  |
| Lorisiformes | **37.5** | 22.1 |  | 31.4-37.8 | 13.8 -14.2 |  | 28.1 |  |  |  |  |  | 30.5 |
| Galagonidae | **18.6** | 1.9 |  | 19.8-23.6 |  |  | 17.9 |  |  |  | 28.3 |  | 19.9 |
| Loridae |  |  | 23 | 16.2-18.0 |  |  |  |  |  |  |  |  | 15.8 |
| Lorinae | **25.9** | 10.4 |  | 9.0-12.0 |  |  |  |  |  |  |  |  | 8.4 |
| Periodicticinae | **18.2** |  |  | 6.1-8.7 |  |  | 6.8 |  |  |  |  |  | 6.2 |
| Lemuriformes | **32.4** | 39.6 | 45 | 67.7-78.5 | 39.6 – 40.7 | 62-65‡ |  |  |  |  |  |  | 76 |
| Indridae | **20.6** | 14 |  |  |  |  |  |  |  |  |  |  | 64.5 |
| Lemuridae | **21.3** | 16.6 |  |  |  |  |  |  |  |  | 23.9 |  | 35.4 |
| Cheirogalidae | **23.6** | 30.7 | 22 |  |  |  |  |  |  |  |  |  |  |
| Megaladapidae |  |  |  |  |  |  |  |  |  |  | 8.8 |  |  |
| Haplorrhini | **42.8** |  | 58 |  | 56.7 – 58.4 |  |  |  |  |  |  |  |  |
| Tarsismiiformes | **14.3** |  |  |  |  |  |  |  |  |  | 9.7 |  | 55.3 |
| Platyrrhini | **26.6** | 28.8 |  |  |  |  |  |  |  |  |  |  |  |
| Cebidae | **18.5** | 32.2 | 22 |  |  |  |  |  |  |  | 23.3 |  | 26.1 |
| Atelidae | **15.2** | 15.1 | 16 |  |  |  |  |  |  |  | 16 |  |  |
| Pitheciidae (Callicebinae) | **26.6** | 9.5 | 17 |  |  |  |  |  |  |  | 9.9 |  |  |
| Catarrhini | **29.3** | 49.6 |  |  |  |  |  |  | 20.8 |  |  |  |  |
| Cercopithecidae | **23.4** | 9.6 | 25 |  |  |  |  |  | 17.1 |  | 21.6 |  | 70.1 |
| Cercopithecidae (Papionini) | **16.1** | 9.2 | 7 |  |  |  |  |  |  |  |  |  |  |
| Cercopithecidae (Cercopithecini) | **15.7** | 5.2 | 14 |  |  |  |  |  |  |  | 17.7 |  |  |
| Cercopithecidae (Macaca) | **11.0** | 5 |  |  |  |  |  |  |  |  |  |  |  |
| Cercopithecidae (Colobinae) | 18.4 | 9.3 | 10 |  |  |  |  |  |  |  | 15.6 |  |  |
| Hominoidea | **21.5** | 17.6 | 18 |  |  |  |  |  |  |  |  |  |  |
| Hylobatidae | **10.3** | 6.3 | 8 |  |  |  |  |  |  |  | 9.4 |  |  |
| Hylobates | **5.5** |  |  |  |  |  |  |  |  |  | 9.4 |  |  |
| Hominidae | **15.9** | 14.5 | 14 |  |  |  |  |  |  |  | 19.7 |  |  |
| Pan | **2.8** | 2.5 |  |  |  |  |  |  |  |  | 3.9 |  |  |
