# Supplementary material for: Estimating the phylogeny and divergence times of primates using a supermatrix approach
Source: BMC Evol Biol. 2009 Oct 27;9:259. doi: 10.1186/1471-2148-9-259 (PMC2774700; doi:10.1186/1471-2148-9-259)
Supplement: Additional file 6 — Tables S3 S4 S5. Table S3. Details of mitochondrial species-level supermatrix. Table S4. Details of mitochondrial genus-level supermatrix. Table S5. Details of nuclear genus-level supermatrix. [file 1471-2148-9-259-S6.DOC]

**Table S3.** Details of mitochondrial species-level supermatrix

| Genus | **Species/ Subspecies** | **Total genes** | ***12s*** | ***16s*** | ***cox2*** | ***cytb*** | ***nd3*** | ***nd4*** | ***nd4l*** |
| --- | --- | --- | --- | --- | --- | --- | --- | --- | --- |
| *Allenopithecus* | *nigroviridis* | **3** | 1 | 1 | 1 |  |  |  |  |
| *Allocebus* | *trichotis* | **4** |  |  |  | 1 | 1 | 1 | 1 |
| ***Alouatta*** | ***belzebul*** | **1** |  |  |  | 1 |  |  |  |
| ***Alouatta*** | ***caraya*** | **2** |  |  | 1 | 1 |  |  |  |
| ***Alouatta*** | ***coibensis*** | **1** |  |  |  | 1 |  |  |  |
| ***Alouatta*** | ***guariba*** | **1** |  |  |  | 1 |  |  |  |
| ***Alouatta*** | ***macconnelli*** | **1** |  |  |  | 1 |  |  |  |
| *Alouatta* | *palliata* | **4** | 1 | 1 | 1 | 1 |  |  |  |
| ***Alouatta*** | ***pigra*** | **1** |  |  |  | 1 |  |  |  |
| ***Alouatta*** | ***sara*** | **1** |  |  |  | 1 |  |  |  |
| *Alouatta* | *seniculus* | **3** | 1 |  | 1 | 1 |  |  |  |
| *Aotus* | *azarae* | **4** | 1 | 1 | 1 | 1 |  |  |  |
| ***Aotus*** | ***lemurinus*** | **2** |  |  | 1 | 1 |  |  |  |
| ***Aotus*** | ***nancymaae*** | **2** |  |  | 1 | 1 |  |  |  |
| ***Aotus*** | ***nigriceps*** | **1** |  |  | 1 |  |  |  |  |
| *Aotus* | *trivirgatus* | **7** | 1 | 1 | 1 | 1 | 1 | 1 | 1 |
| ***Aotus*** | ***vociferans*** | **1** |  |  | 1 |  |  |  |  |
| ***Arctocebus*** | ***aureus*** | **2** | 1 | 1 |  |  |  |  |  |
| ***Arctocebus*** | ***calabarensis*** | **2** | 1 |  |  | 1 |  |  |  |
| ***Ateles*** | ***chamek*** | **1** |  |  | 1 |  |  |  |  |
| *Ateles* | *fusciceps* | **3** | 1 | 1 | 1 |  |  |  |  |
| *Ateles* | *geoffroyi* | **4** |  | 1 | 1 | 1 |  | 1 |  |
| ***Ateles*** | ***marginatus*** | **1** |  |  | 1 |  |  |  |  |
| ***Ateles*** | ***paniscus*** | **1** |  |  | 1 |  |  |  |  |
| *Avahi* | *laniger* | **6** | 1 |  | 1 | 1 | 1 | 1 | 1 |
| *Avahi* | *occidentalis* | **6** | 1 |  | 1 | 1 | 1 | 1 | 1 |
| *Brachyteles* | *arachnoides* | **4** | 1 | 1 | 1 | 1 |  |  |  |
| ***Brachyteles*** | ***hypoxanthus*** | **1** |  |  | 1 |  |  |  |  |
| ***Cacajao*** | ***melanocephalus*** | **1** |  |  |  | 1 |  |  |  |
| ***Callicebus*** | ***hoffmannsi*** | **1** |  |  |  | 1 |  |  |  |
| *Callicebus* | *moloch* | **3** | 1 | 1 |  | 1 |  |  |  |
| ***Callicebus*** | ***personatus*** | **1** |  |  |  | 1 |  |  |  |
| ***Callicebus*** | ***torquatus*** | **1** |  |  |  | 1 |  |  |  |
| *Callimico* | *goeldii* | **5** | 1 | 1 | 1 | 1 |  | 1 |  |
| ***Callithrix*** | ***argentata*** | **2** |  |  | 1 | 1 |  |  |  |
| ***Callithrix*** | ***aurita*** | **1** |  |  | 1 |  |  |  |  |
| ***Callithrix*** | ***emiliae*** | **2** |  |  | 1 | 1 |  |  |  |
| ***Callithrix*** | ***geoffroyi*** | **2** |  |  | 1 | 1 |  |  |  |
| ***Callithrix*** | ***humeralifera*** | **2** |  |  | 1 | 1 |  |  |  |
| *Callithrix* | *jacchus* | **5** | 1 | 1 | 1 | 1 |  | 1 |  |
| ***Callithrix*** | ***mauesi*** | **2** |  |  | 1 | 1 |  |  |  |
| ***Callithrix*** | ***penicillata*** | **2** |  |  | 1 | 1 |  |  |  |
| *Callithrix* | *pygmaea* | **5** | 1 | 1 | 1 | 1 |  | 1 |  |
| *Cebus* | *albifrons* | **7** | 1 | 1 | 1 | 1 | 1 | 1 | 1 |
| *Cebus* | *apella* | **5** | 1 | 1 | 1 | 1 |  | 1 |  |
| ***Cebus*** | ***capucinus*** | **2** |  |  | 1 | 1 |  |  |  |
| ***Cebus*** | ***libidinosus*** | **1** |  |  | 1 |  |  |  |  |
| ***Cebus*** | ***nigritus*** | **1** |  |  | 1 |  |  |  |  |
| *Cercocebus* | *agilis* | **3** | 1 | 1 | 1 |  |  |  |  |
| ***Cercocebus*** | ***atys*** | **1** |  |  | 1 |  |  |  |  |
| *Cercocebus* | *torquatus* | **4** | 1 |  | 1 | 1 |  | 1 |  |
| ***Cercopithecus*** | ***ascanius*** | **2** | 1 | 1 |  |  |  |  |  |
| ***Cercopithecus*** | ***campbelli*** | **2** | 1 | 1 |  |  |  |  |  |
| *Cercopithecus* | *cephus* | **3** | 1 | 1 | 1 |  |  |  |  |
| ***Cercopithecus*** | ***erythrogaster*** | **2** | 1 | 1 |  |  |  |  |  |
| ***Cercopithecus*** | ***erythrotis*** | **2** | 1 | 1 |  |  |  |  |  |
| ***Cercopithecus*** | ***hamlyni*** | **2** | 1 | 1 |  |  |  |  |  |
| *Cercopithecus* | *lhoesti* | **3** | 1 | 1 | 1 |  |  |  |  |
| ***Cercopithecus*** | ***mitis*** | **2** | 1 |  | 1 |  |  |  |  |
| *Cercopithecus* | *mona* | **4** | 1 | 1 | 1 | 1 |  |  |  |
| ***Cercopithecus*** | ***neglectus*** | **2** | 1 |  | 1 |  |  |  |  |
| *Cercopithecus* | *nictitans* | **4** | 1 | 1 | 1 | 1 |  |  |  |
| ***Cercopithecus*** | ***petaurista*** | **2** | 1 | 1 |  |  |  |  |  |
| ***Cercopithecus*** | ***pogonias*** | **1** |  | 1 |  |  |  |  |  |
| *Cercopithecus* | *preussi* | **3** | 1 | 1 | 1 |  |  |  |  |
| ***Cercopithecus*** | ***roloway*** | **1** | 1 |  |  |  |  |  |  |
| ***Cercopithecus*** | ***solatus*** | **2** | 1 | 1 |  |  |  |  |  |
| ***Cheirogaleus*** | ***crossleyi*** | **1** |  |  |  | 1 |  |  |  |
| *Cheirogaleus* | *major* | **7** | 1 | 1 | 1 | 1 | 1 | 1 | 1 |
| *Cheirogaleus* | *medius* | **7** | 1 | 1 | 1 | 1 | 1 | 1 | 1 |
| ***Chiropotes*** | ***albinasus*** | **2** | 1 | 1 |  |  |  |  |  |
| ***Chiropotes*** | ***satanas*** | **1** | 1 |  |  |  |  |  |  |
| *Chlorocebus* | *aethiops* | **6** | 1 | 1 | 1 | 1 | 1 |  | 1 |
| *Chlorocebus* | *pygerythrus* | **7** | 1 | 1 | 1 | 1 | 1 | 1 | 1 |
| *Chlorocebus* | *sebaeus* | **7** | 1 | 1 | 1 | 1 | 1 | 1 | 1 |
| *Chlorocebus* | *tantalus* | **7** | 1 | 1 | 1 | 1 | 1 | 1 | 1 |
| *Colobus* | *angolensis* | **4** |  |  |  | 1 | 1 | 1 | 1 |
| *Colobus* | *badius* | **4** | 1 | 1 | 1 | 1 |  |  |  |
| *Colobus* | *guereza* | **7** | 1 | 1 | 1 | 1 | 1 | 1 | 1 |
| *Colobus* | *polykomos* | **4** |  |  |  | 1 | 1 | 1 | 1 |
| *Cynocephalus* | *variegatus* | **7** | 1 | 1 | 1 | 1 | 1 | 1 | 1 |
| *Daubentonia* | *madagascariensis* | **7** | 1 | 1 | 1 | 1 | 1 | 1 | 1 |
| *Erythrocebus* | *patas* | **4** | 1 | 1 | 1 |  |  | 1 |  |
| *Eulemur* | *albifrons* | **7** | 1 | 1 | 1 | 1 | 1 | 1 | 1 |
| *Eulemur* | *albocollaris* | **5** | 1 |  |  | 1 | 1 | 1 | 1 |
| *Eulemur* | *collaris* | **7** | 1 | 1 | 1 | 1 | 1 | 1 | 1 |
| *Eulemur* | *coronatus* | **6** | 1 | 1 |  | 1 | 1 | 1 | 1 |
| *Eulemur* | *fulvus* | **5** | 1 |  |  | 1 | 1 | 1 | 1 |
| *Eulemur* | *macaco* | **7** | 1 | 1 | 1 | 1 | 1 | 1 | 1 |
| *Eulemur* | *mongoz* | **7** | 1 | 1 | 1 | 1 | 1 | 1 | 1 |
| *Eulemur* | *rubriventer* | **6** | 1 |  | 1 | 1 | 1 | 1 | 1 |
| *Eulemur* | *rufus* | **7** | 1 | 1 | 1 | 1 | 1 | 1 | 1 |
| *Eulemur* | *sanfordi* | **5** | 1 |  |  | 1 | 1 | 1 | 1 |
| *Euoticus* | *elegantulus* | **3** | 1 | 1 |  | 1 |  |  |  |
| *Galago* | *alleni* | **3** | 1 | 1 |  | 1 |  |  |  |
| *Galago* | *demidoff* | **4** | 1 | 1 | 1 | 1 |  |  |  |
| ***Galago*** | ***gabonensis*** | **1** |  |  |  | 1 |  |  |  |
| *Galago* | *gallarum* | **3** | 1 | 1 |  | 1 |  |  |  |
| ***Galago*** | ***granti*** | **2** | 1 |  |  | 1 |  |  |  |
| *Galago* | *moholi* | **3** | 1 | 1 |  | 1 |  |  |  |
| *Galago* | *senegalensis* | **7** | 1 | 1 | 1 | 1 | 1 | 1 | 1 |
| *Galago* | *zanzibaricus* | **3** | 1 | 1 |  | 1 |  |  |  |
| *Gorilla* | *gorilla* | **7** | 1 | 1 | 1 | 1 | 1 | 1 | 1 |
| *Hapalemur* | *alaotrensis* | **5** | 1 |  |  | 1 | 1 | 1 | 1 |
| *Hapalemur* | *aureus* | **6** | 1 |  | 1 | 1 | 1 | 1 | 1 |
| *Hapalemur* | *griseus* | **7** | 1 | 1 | 1 | 1 | 1 | 1 | 1 |
| *Hapalemur* | *occidentalis* | **6** | 1 |  | 1 | 1 | 1 | 1 | 1 |
| *Hapalemur* | *simus* | **6** | 1 |  | 1 | 1 | 1 | 1 | 1 |
| *Homo* | *sapiens* | **7** | 1 | 1 | 1 | 1 | 1 | 1 | 1 |
| *Hoolock* | *hoolock* | **4** |  |  |  | 1 | 1 | 1 | 1 |
| *Hylobates* | *agilis* | **6** | 1 | 1 |  | 1 | 1 | 1 | 1 |
| *Hylobates* | *klossii* | **6** | 1 | 1 |  | 1 | 1 | 1 | 1 |
| *Hylobates* | *lar* | **7** | 1 | 1 | 1 | 1 | 1 | 1 | 1 |
| *Hylobates* | *moloch* | **6** | 1 | 1 |  | 1 | 1 | 1 | 1 |
| *Hylobates* | *muelleri* | **6** | 1 | 1 |  | 1 | 1 | 1 | 1 |
| *Hylobates* | *pileatus* | **6** |  | 1 | 1 | 1 | 1 | 1 | 1 |
| *Indri* | *indri* | **5** | 1 |  |  | 1 | 1 | 1 | 1 |
| *Lagothrix* | *lagotricha* | **4** | 1 | 1 | 1 | 1 |  |  |  |
| *Lemur* | *catta* | **7** | 1 | 1 | 1 | 1 | 1 | 1 | 1 |
| ***Leontopithecus*** | ***chrysomelas*** | **2** |  |  |  | 1 |  | 1 |  |
| ***Leontopithecus*** | ***chrysopygus*** | **1** |  |  |  | 1 |  |  |  |
| *Leontopithecus* | *rosalia* | **4** | 1 | 1 |  | 1 |  | 1 |  |
| *Lepilemur* | *dorsalis* | **5** | 1 |  |  | 1 | 1 | 1 | 1 |
| *Lepilemur* | *edwardsi* | **5** | 1 |  |  | 1 | 1 | 1 | 1 |
| *Lepilemur* | *leucopus* | **5** | 1 |  |  | 1 | 1 | 1 | 1 |
| *Lepilemur* | *mustelinus* | **5** | 1 |  |  | 1 | 1 | 1 | 1 |
| *Lepilemur* | *ruficaudatus* | **6** | 1 |  | 1 | 1 | 1 | 1 | 1 |
| *Lepilemur* | *septentrionalis* | **6** | 1 |  | 1 | 1 | 1 | 1 | 1 |
| *Lophocebus* | *albigena* | **4** | 1 | 1 | 1 | 1 |  |  |  |
| ***Lophocebus*** | ***atterimus*** | **2** | 1 |  | 1 |  |  |  |  |
| *Loris* | *lydekkerianus* | **3** | 1 | 1 |  | 1 |  |  |  |
| *Loris* | *tardigradus* | **4** | 1 | 1 | 1 | 1 |  |  |  |
| *Macaca* | *arctoides* | **5** | 1 | 1 | 1 | 1 |  | 1 |  |
| *Macaca* | *assamensis* | **5** | 1 | 1 | 1 | 1 |  | 1 |  |
| *Macaca* | *cyclopis* | **4** | 1 | 1 | 1 | 1 |  |  |  |
| *Macaca* | *fascicularis* | **6** | 1 | 1 | 1 | 1 |  | 1 | 1 |
| *Macaca* | *fuscata* | **4** | 1 | 1 | 1 |  |  | 1 |  |
| *Macaca* | *hecki* | **5** | 1 | 1 |  | 1 |  | 1 | 1 |
| *Macaca* | *leonina* | **5** | 1 |  | 1 | 1 |  | 1 | 1 |
| *Macaca* | *maurus* | **4** | 1 | 1 |  | 1 |  |  | 1 |
| *Macaca* | *mulatta* | **7** | 1 | 1 | 1 | 1 | 1 | 1 | 1 |
| *Macaca* | *nemestrina* | **6** | 1 | 1 | 1 | 1 |  | 1 | 1 |
| *Macaca* | *nigra* | **5** | 1 | 1 |  | 1 |  | 1 | 1 |
| *Macaca* | *nigrescens* | **5** | 1 | 1 |  | 1 |  | 1 | 1 |
| *Macaca* | *ochreata* | **5** | 1 | 1 |  | 1 |  | 1 | 1 |
| *Macaca* | *pagensis* | **5** | 1 | 1 |  | 1 |  | 1 | 1 |
| *Macaca* | *radiata* | **4** | 1 | 1 | 1 |  |  | 1 |  |
| *Macaca* | *silenus* | **5** | 1 | 1 | 1 | 1 |  | 1 |  |
| *Macaca* | *sinica* | **3** | 1 | 1 |  |  |  | 1 |  |
| *Macaca* | *sylvanus* | **7** | 1 | 1 | 1 | 1 | 1 | 1 | 1 |
| *Macaca* | *thibetana* | **5** | 1 | 1 | 1 | 1 |  | 1 |  |
| *Macaca* | *tonkeana* | **5** | 1 | 1 |  | 1 |  | 1 | 1 |
| ***Mandrillus*** | ***leucophaeus*** | **2** |  |  | 1 | 1 |  |  |  |
| *Mandrillus* | *sphinx* | **5** | 1 | 1 | 1 | 1 |  | 1 |  |
| *Microcebus* | *murinus* | **7** | 1 | 1 | 1 | 1 | 1 | 1 | 1 |
| ***Microcebus*** | ***myoxinus*** | **2** |  |  | 1 | 1 |  |  |  |
| *Microcebus* | *ravelobensis* | **6** | 1 |  | 1 | 1 | 1 | 1 | 1 |
| *Microcebus* | *rufus* | **6** | 1 |  | 1 | 1 | 1 | 1 | 1 |
| ***Miopithecus*** | ***ougouensis*** | **1** | 1 |  |  |  |  |  |  |
| ***Miopithecus*** | ***talapoin*** | **1** | 1 |  |  |  |  |  |  |
| *Mirza* | *coquereli* | **7** | 1 | 1 | 1 | 1 | 1 | 1 | 1 |
| *Nasalis* | *larvatus* | **7** | 1 | 1 | 1 | 1 | 1 | 1 | 1 |
| *Nomascus* | *concolor* | **5** | 1 | 1 |  | 1 |  | 1 | 1 |
| *Nomascus* | *gabriellae* | **4** |  |  |  | 1 | 1 | 1 | 1 |
| *Nomascus* | *leucogenys* | **5** |  |  | 1 | 1 | 1 | 1 | 1 |
| ***Nycticebus*** | ***bengalensis*** | **1** |  |  |  | 1 |  |  |  |
| *Nycticebus* | *coucang* | **7** | 1 | 1 | 1 | 1 | 1 | 1 | 1 |
| *Nycticebus* | *pygmaeus* | **3** | 1 | 1 |  | 1 |  |  |  |
| *Otolemur* | *crassicaudatus* | **6** | 1 | 1 |  | 1 | 1 | 1 | 1 |
| *Otolemur* | *garnetti* | **3** | 1 | 1 |  | 1 |  |  |  |
| *Pan* | *paniscus* | **7** | 1 | 1 | 1 | 1 | 1 | 1 | 1 |
| *Pan* | *troglodytes* | **7** | 1 | 1 | 1 | 1 | 1 | 1 | 1 |
| *Papio* | *anubis* | **3** |  |  | 1 | 1 |  | 1 |  |
| *Papio* | *cynocephalus* | **4** | 1 |  | 1 | 1 |  | 1 |  |
| *Papio* | *hamadryas* | **7** | 1 | 1 | 1 | 1 | 1 | 1 | 1 |
| ***Papio*** | ***papio*** | **2** |  |  | 1 |  |  | 1 |  |
| *Papio* | *ursinus* | **3** | 1 |  | 1 |  |  | 1 |  |
| *Perodicticus* | *potto* | **3** | 1 | 1 |  | 1 |  |  |  |
| ***Phaner*** | ***furcifer*** | **1** |  |  |  | 1 |  |  |  |
| ***Pithecia*** | ***irrorata*** | **1** |  |  |  | 1 |  |  |  |
| ***Pithecia*** | ***pithecia*** | **2** | 1 | 1 |  |  |  |  |  |
| *Pongo* | *abelii* | **7** | 1 | 1 | 1 | 1 | 1 | 1 | 1 |
| *Pongo* | *pygmaeus* | **7** | 1 | 1 | 1 | 1 | 1 | 1 | 1 |
| ***Presbytis*** | ***comata*** | **1** |  |  |  | 1 |  |  |  |
| *Presbytis* | *melalophos* | **7** | 1 | 1 | 1 | 1 | 1 | 1 | 1 |
| *Propithecus* | *coquereli* | **6** |  | 1 | 1 | 1 | 1 | 1 | 1 |
| *Propithecus* | *deckenii* | **3** |  |  |  |  | 1 | 1 | 1 |
| *Propithecus* | *diadema* | **7** | 1 | 1 | 1 | 1 | 1 | 1 | 1 |
| *Propithecus* | *tattersalli* | **6** | 1 |  | 1 | 1 | 1 | 1 | 1 |
| *Propithecus* | *verreauxi* | **7** | 1 | 1 | 1 | 1 | 1 | 1 | 1 |
| *Pygathrix* | *nemaeus* | **7** | 1 | 1 | 1 | 1 | 1 | 1 | 1 |
| ***Rhinopithecus*** | ***avunculus*** | **2** |  | 1 |  | 1 |  |  |  |
| Rhinopithecus | *bieti* | **5** | 1 |  |  | 1 | 1 | 1 | 1 |
| ***Rhinopithecus*** | ***brelichi*** | **2** | 1 |  |  | 1 |  |  |  |
| *Rhinopithecus* | *roxellana* | **7** | 1 | 1 | 1 | 1 | 1 | 1 | 1 |
| ***Saguinus*** | ***fuscicollis*** | **1** |  |  |  | 1 |  |  |  |
| *Saguinus* | *geoffroyi* | **3** | 1 | 1 |  | 1 |  |  |  |
| ***Saguinus*** | ***melanoleucus*** | **1** |  |  |  | 1 |  |  |  |
| ***Saguinus*** | ***midas*** | **2** |  |  |  | 1 |  | 1 |  |
| ***Saguinus*** | ***oedipus*** | **2** | 1 |  |  | 1 |  |  |  |
| *Saimiri* | *boliviensis* | **3** |  | 1 | 1 | 1 |  |  |  |
| ***Saimiri*** | ***oerstedti*** | **1** |  |  |  | 1 |  |  |  |
| *Saimiri* | *sciureus* | **5** | 1 | 1 | 1 | 1 |  | 1 |  |
| *Semnopithecus* | *entellus* | **7** | 1 | 1 | 1 | 1 | 1 | 1 | 1 |
| ***Semnopithecus*** | ***johnii*** | **1** |  |  |  | 1 |  |  |  |
| ***Semnopithecus*** | ***vetulus*** | **1** |  |  |  | 1 |  |  |  |
| ***Simias*** | ***concolor*** | **1** |  |  |  | 1 |  |  |  |
| *Symphalangus* | *syndactylus* | **7** | 1 | 1 | 1 | 1 | 1 | 1 | 1 |
| *Tarsius* | *bancanus* | **7** | 1 | 1 | 1 | 1 | 1 | 1 | 1 |
| *Tarsius* | *syrichta* | **4** | 1 | 1 | 1 |  |  | 1 |  |
| *Theropithecus* | *gelada* | **6** | 1 | 1 | 1 |  | 1 | 1 | 1 |
| ***Trachypithecus*** | ***auratus*** | **1** |  |  |  | 1 |  |  |  |
| ***Trachypithecus*** | ***barbei*** | **1** |  |  |  | 1 |  |  |  |
| *Trachypithecus* | *francoisi* | **5** |  |  | 1 | 1 | 1 | 1 | 1 |
| ***Trachypithecus*** | ***geei*** | **1** |  |  |  | 1 |  |  |  |
| *Trachypithecus* | *obscurus* | **7** | 1 | 1 | 1 | 1 | 1 | 1 | 1 |
| ***Trachypithecus*** | ***pileatus*** | **1** |  |  |  | 1 |  |  |  |
| *Varecia* | *rubra* | **6** | 1 |  | 1 | 1 | 1 | 1 | 1 |
| *Varecia* | *variegata* | **7** | 1 | 1 | 1 | 1 | 1 | 1 | 1 |

**Table S4.** Details of mitochondrial genus-level supermatrix

| **Genus** | **Species/ subspecies** | **Total genes** | ***12s*** | ***16s*** | ***cox2*** | ***cytb*** | ***nd3*** | ***nd4*** | ***nd4l*** | **Chimaeric sequences** |
| --- | --- | --- | --- | --- | --- | --- | --- | --- | --- | --- |
| Allenopithecus | *nigroviridis* | 3 | 1 | 1 | 1 |  |  |  |  |  |
| *Allocebus* | *trichotis* | 4 |  |  |  | 1 | 1 | 1 | 1 |  |
| *Alouatta* | *palliata* | 4 | 1 | 1 | 1 | 1 |  |  |  |  |
| *Aotus* | *trivirgatus* | 7 | 1 | 1 | 1 | 1 | 1 | 1 | 1 |  |
| ***Arctocebus*** | ***aureus*** | 3 | 1 | 1 |  | 1 |  |  |  | *cytb* from *calabarensis* |
| *Ateles* | *geoffroyi* | 5 | 1 | 1 | 1 | 1 |  | 1 |  | *12s* from *fusciceps* |
| *Avahi* | *laniger* | 6 | 1 |  | 1 | 1 | 1 | 1 | 1 |  |
| *Brachyteles* | *arachnoides* | 4 | 1 | 1 | 1 | 1 |  |  |  |  |
| *Hoolock* | *hoolock* | 4 |  |  |  | 1 | 1 | 1 | 1 |  |
| ***Cacajao*** | ***melanocephalus*** | 1 |  |  |  | 1 |  |  |  |  |
| *Callicebus* | *moloch* | 3 | 1 | 1 |  | 1 |  |  |  |  |
| *Callimico* | *goeldii* | 5 | 1 | 1 | 1 | 1 |  | 1 |  |  |
| *Callithrix* | *jacchus* | 5 | 1 | 1 | 1 | 1 |  | 1 |  |  |
| *Cebus* | *albifrons* | 7 | 1 | 1 | 1 | 1 | 1 | 1 | 1 |  |
| *Cheirogaleus* | *major* | 7 | 1 | 1 | 1 | 1 | 1 | 1 | 1 |  |
| ***Chiropotes*** | ***albinasus*** | 2 | 1 | 1 |  |  |  |  |  |  |
| *Chlorocebus* | *pygerythrus* | 7 | 1 | 1 | 1 | 1 | 1 | 1 | 1 |  |
| *Colobus* | *guereza* | 7 | 1 | 1 | 1 | 1 | 1 | 1 | 1 |  |
| *Cynocephalus* | *variegatus* | 7 | 1 | 1 | 1 | 1 | 1 | 1 | 1 |  |
| *Daubentonia* | *madagascariensis* | 7 | 1 | 1 | 1 | 1 | 1 | 1 | 1 |  |
| *Eulemur* | *albifrons* | 7 | 1 | 1 | 1 | 1 | 1 | 1 | 1 |  |
| *Galago* | *senegalensis* | 7 | 1 | 1 | 1 | 1 | 1 | 1 | 1 |  |
| *Gorilla* | *gorilla* | 7 | 1 | 1 | 1 | 1 | 1 | 1 | 1 |  |
| *Hapalemur* | *griseus* | 7 | 1 | 1 | 1 | 1 | 1 | 1 | 1 |  |
| *Homo* | *sapiens* | 7 | 1 | 1 | 1 | 1 | 1 | 1 | 1 |  |
| *Hylobates* | *lar* | 7 | 1 | 1 | 1 | 1 | 1 | 1 | 1 |  |
| *Indri* | *indri* | 5 | 1 |  |  | 1 | 1 | 1 | 1 |  |
| *Lagothrix* | *lagotricha* | 4 | 1 | 1 | 1 | 1 |  |  |  |  |
| *Lemur* | *catta* | 7 | 1 | 1 | 1 | 1 | 1 | 1 | 1 |  |
| *Leontopithecus* | *rosalia* | 4 | 1 | 1 |  | 1 |  | 1 |  |  |
| *Lepilemur* | *ruficaudatus* | 6 | 1 |  | 1 | 1 | 1 | 1 | 1 |  |
| *Lophocebus* | *albigena* | 4 | 1 | 1 | 1 | 1 |  |  |  |  |
| *Loris* | *tardigradus* | 4 | 1 | 1 | 1 | 1 |  |  |  |  |
| *Macaca* | *mulatta* | 7 | 1 | 1 | 1 | 1 | 1 | 1 | 1 |  |
| *Mandrillus* | *sphinx* | 5 | 1 | 1 | 1 | 1 |  | 1 |  |  |
| *Microcebus* | *murinus* | 7 | 1 | 1 | 1 | 1 | 1 | 1 | 1 |  |
| ***Miopithecus*** | ***ougouensis*** | 1 | 1 |  |  |  |  |  |  |  |
| *Mirza* | *coquereli* | 7 | 1 | 1 | 1 | 1 | 1 | 1 | 1 |  |
| *Nasalis* | *larvatus* | 7 | 1 | 1 | 1 | 1 | 1 | 1 | 1 |  |
| Nomascus | *concolor* | 7 | 1 | 1 | 1 | 1 | 1 | 1 | 1 | *cox2*, *nd3* from *leucogenys* |
| *Nycticebus* | *coucang* | 7 | 1 | 1 | 1 | 1 | 1 | 1 | 1 |  |
| *Pan* | *paniscus* | 7 | 1 | 1 | 1 | 1 | 1 | 1 | 1 |  |
| *Papio* | *hamadryas* | 7 | 1 | 1 | 1 | 1 | 1 | 1 | 1 |  |
| *Perodicticus* | *potto* | 3 | 1 | 1 |  | 1 |  |  |  |  |
| ***Phaner*** | ***furcifer*** | 1 |  |  |  | 1 |  |  |  |  |
| ***Pithecia*** | ***pithecia*** | 3 | 1 | 1 |  | 1 |  |  |  | *cytb* from *irrorata* |
| *Pongo* | *abelii* | 7 | 1 | 1 | 1 | 1 | 1 | 1 | 1 |  |
| *Presbytis* | *melalophos* | 7 | 1 | 1 | 1 | 1 | 1 | 1 | 1 |  |
| *Propithecus* | *diadema* | 7 | 1 | 1 | 1 | 1 | 1 | 1 | 1 |  |
| *Pygathrix* | *nemaeus* | 7 | 1 | 1 | 1 | 1 | 1 | 1 | 1 |  |
| *Rhinopithecus* | *roxellana* | 7 | 1 | 1 | 1 | 1 | 1 | 1 | 1 |  |
| *Saguinus* | *geoffroyi* | 4 | 1 | 1 |  | 1 |  | 1 |  | *nd4* from *midas* |
| *Saimiri* | *sciureus* | 5 | 1 | 1 | 1 | 1 |  | 1 |  |  |
| ***Simias*** | ***concolor*** | 1 |  |  |  | 1 |  |  |  |  |
| *Symphalangus* | *syndactylus* | 7 | 1 | 1 | 1 | 1 | 1 | 1 | 1 |  |
| *Tarsius* | *bancanus* | 7 | 1 | 1 | 1 | 1 | 1 | 1 | 1 |  |
| *Theropithecus* | *gelada* | 6 | 1 | 1 | 1 |  | 1 | 1 | 1 |  |
| *Varecia* | *variegata* | 7 | 1 | 1 | 1 | 1 | 1 | 1 | 1 |  |

**Table S5.** Details of nuclear genus-level supermatrix

| Genus | Species/ subspecies | Total genes | *cxcr4* | ***SRY*** | ***TSPY*** | **Chimaeric sequences** |
| --- | --- | --- | --- | --- | --- | --- |
| *Allenopithecus* | *nigroviridis* | 2 |  | 1 | 1 |  |
| *Alouatta* | *palliata* | 2 | 1 | 1 |  | *cxcr4* from *seniculus* |
| *Aotus* | *azarae* | 2 |  | 1 | 1 | *tspy* from *trivirgatus* |
| *Callimico* | *goeldii* | 2 | 1 | 1 |  |  |
| *Callithrix* | *jacchus* | 2 | 1 | 1 |  |  |
| *Cebus* | *apella* | 2 |  | 1 | 1 |  |
| *Chlorocebus* | *aethiops* | 3 | 1 | 1 | 1 |  |
| *Erythrocebus* | *patas* | 2 |  | 1 | 1 |  |
| *Gorilla* | *gorilla* | 2 | 1 | 1 |  |  |
| *Homo* | *sapiens* | 3 | 1 | 1 | 1 |  |
| *Hylobates* | *lar* | 2 | 1 | 1 |  |  |
| *Leontopithecus* | *chrysomelas* | 1 |  | 1 |  |  |
| *Lophocebus* | *albigena* | 2 |  | 1 | 1 |  |
| *Macaca* | *arctoides* | 3 | 1 | 1 | 1 |  |
| *Mandrillus* | *sphinx* | 3 | 1 | 1 | 1 |  |
| *Miopithecus* | *talapoin* | 2 |  | 1 | 1 |  |
| *Pan* | *troglodytes* | 3 | 1 | 1 | 1 |  |
| *Papio* | *hamadryas* | 3 | 1 | 1 | 1 | *cxcr4* from *anubis* |
| *Pongo* | *pygmaeus* | 2 | 1 | 1 |  |  |
| *Presbytis* | *melalophos* | 2 |  | 1 | 1 |  |
| *Pygathrix* | *nemaeus* | 2 | 1 | 1 |  |  |
| *Rhinopithecus* | *bieti* | 2 | 1 | 1 |  |  |
| *Saguinus* | *midas* | 2 | 1 | 1 |  | *cxcr4* from *oedipus* |
| *Saimiri* | *sciureus* | 3 | 1 | 1 | 1 |  |
| *Theropithecus* | *gelada* | 2 |  | 1 | 1 |  |
| *Trachypithecus* | *auratus* | 2 | 1 | 1 |  | *cxcr4* from *francoisi* |
