# Supplementary material for: Estimating the phylogeny and divergence times of primates using a supermatrix approach
Source: BMC Evol Biol. 2009 Oct 27;9:259. doi: 10.1186/1471-2148-9-259 (PMC2774700; doi:10.1186/1471-2148-9-259)
Supplement: Additional file 7 — Table S6. Primate fossil evidence used to calibrate phylogenetic estimates of divergence times. Times are taken from Hartwig (2002). The earliest fossil evidence for the base of each group is used to place a minimum age constraint on its parent node in the tree. Minimum and maximum age constraints of 64 to 110 MYA, respectively, were also specified for the root node (divergence between primates and flying lemur). [file 1471-2148-9-259-S7.DOC]

## Table S6 - Primate fossil evidence used to calibrate phylogenetic estimates of divergence times

Times are taken from Hartwig (2002). The earliest fossil evidence for the base of each group is used to place a minimum age constraint on its parent node in the tree. Minimum and maximum age constraints of 64 and 110 MYA, respectively, were also specified for the root node (divergence between primates and flying lemur).

| **Group** | **Fossil(s)** | **Horizon^a^** | **Minimum age (MYA)** |
| --- | --- | --- | --- |
| Strepsirrhini | *Cantius, Donrussellia, Protoadapis* | E. Eocene | 50.0 |
| ?Chiromyiformes | *Plesiopithecus teras* | L. Eocene | 35.6 |
| ?Lorisidae | *Karanisia clarki* | L. M. Eocene | 36.9 |
| Cebidae,?Saimiriini | *Dolichocebus gaimanensis* | E. Miocene | 20.5 |
| Alouattinae | *Stirtonia spp.* | L. M. Miocene | 12.6 |
| Saimiriinae | *Neosaimiri fieldsi* | L. M. Miocene | 12.1 |
| Catarrhini | *Catopithecus browni* | L. Eocene | 35.6^b^ |
| Colobinae | *Microcolobus tugenensis* | L. Miocene | 10.0 |
| *Macaca sylvanus* group | *Macaca libyca* | L. Miocene | 6.0 |
| Hominoidea | *Morotopithecus bishopi* | E. Miocene | 20.6^c^ |
| Homininae | *Dryopithecus fontani* | L. Miocene | 11.0 |
| Gorillini | *Chororapithecus abyssinicus* | L. Miocene | 10.0 |
| Hominini | *Orrorin tugenensis* | L. Miocene | 5.9 |

^a^E.=Early, M.=Middle, L.=Late.

^b^This calibration was used to specify an exponential prior, with a minimum bound of 35.6 Myr and an upper 97.5% soft bound of 42 Myr.

^c^This calibration was used to specify an exponential prior, with a minimum bound of 20.6 Myr and an upper 97.5% soft bound of 30 Myr.
